# Theoretical Investigation of Lorentz Transformation of Relativistic Quantities in Two-Dimensional Spacetime Continuum

**DOI:** 10.1155/tswj/7149569

**Published:** 2025-05-15

**Authors:** Chandra Bahadur Khadka, Bhishma Karki

**Affiliations:** ^1^Department of Physics, Tri-Chandra Multiple Campus, Tribhuvan University, Kathmandu, Nepal; ^2^National Research Council Nepal, Kathmandu, Nepal

**Keywords:** four-vector, Lorentz invariance, Lorentz transformation equations, special relativity

## Abstract

In the current study, we conducted a theoretical study to derive the Lorentz transformation between inertial frames of reference moving in two-dimensional spacetime continuum. The invariance of the space–time interval equation, with use of the derived two-dimensional transformations, yields the notion of 2 + 2-dimensional spacetime continuum which consists of two space and two time coordinates. The velocity addition formulas, Lorentz transformations of energy and momentum are then investigated in 2 + 2-dimensional spacetime frame. Additionally, we investigated the concept of four-vector in 2 + 2 dimensions and further discussed their transformation based on the matrix equation, which is fully consistent with the Lorentz invariant energy–momentum relation.

## 1. Introduction

In the framework of special theory of relativity, the Lorentz transformation is a mathematical description of how space and time coordinates are transformed between two inertial frames of reference moving relative to each other at a constant velocity. The mathematical form of one-dimensional Lorentz transformation equations between frames of reference moving along the *x*-axis is given by the relations:

(1) $$\begin{array}{c} \left\{\begin{array}{cc} t^{\prime }=\frac{t-vx/c^{2}}{\sqrt{1-v^{2}/c^{2}}}, & x^{\prime }=\frac{x-vt}{\sqrt{1-v^{2}/c^{2}}}\\ y^{\prime }=y, & z^{\prime }=z \end{array}\right.. \end{array}$$

In 1887, analogous transformations were deduced by Voigt [1] related to an incompressible medium, and these equations were further analyzed by Larmor [2] and Lorentz [3] and finally brought into their modern form by Poincare [4] who gave the transformation the name of Lorentz transformation. In 1905, Einstein [5] obtained Transformation (1) from the principle of relativity and constant light velocity alone by modifying the traditional concept of space and time, without requiring a mechanical ether. Minkowski used Transformation (1) to argue that space and time are inseparably connected as spacetime and introduced the notion of four-vector, that is, the concept of four-dimensional spacetime continuum based on the invariance of square of spacetime interval equation:

(2) $$\begin{array}{c} x^{2}+y^{2}+z^{2}+{\left(ict\right)}^{2}={x^{\prime }}^{2}+{y^{\prime }}^{2}+{z^{\prime }}^{2}+{\left(ict^{\prime }\right)}^{2}. \end{array}$$

Minkowski inspected Equation (2) and made a mental jump to point out that any event with spacetime coordinate (*x*, *y*, *z*, *ict*) in one inertial reference frame appears with coordinate (*x*′, *y*′, *z*′, *ict*′) in another inertial frame. The time coordinate *ict* must be a vector quantity as it contains velocity of light *c* which is obviously a vector quantity by its definition. Thus, time coordinate *ict* may have different values in the *X*, *Y*, and *Z*-directions. Unlike it, Minkowski had obtained only one time coordinate from Equation (2) as the invariance of the Equation (2) was interpreted under one-dimensional Lorentz transformation Equation (1). The invariance of spacetime interval Equation (2) under two-dimensional (2D) Lorentz transformations, that is, the transformation equations between frames of reference moving in the two dimensions of space, is yet to be investigated. In this work, we will introduce the concept of 2 + 2-dimensional spacetime continuum to give the correct matrix form of the derived 2D Lorentz transformation equations between inertial frames of reference moving in 2D *XY*-plane with constant velocity. The main goal is to introduce two time coordinates if the relative motion between frames of reference occurs in a 2D *XY*-plane.

Theoretical investigation on multidimensional time coordinates has been done previously by many researchers. Most of the authors in the works [6–10] present multidimensional time in order to give better explanations of quantum mechanics and the spin. Recami and Mignani [11], Teli [12], Pappas [13], Guy [14], and Weinberg [15] added two extra time coordinates to the four-dimensional spacetime coordinates to interpret the imaginary quantities in the superluminal Lorentz transformations. In Reference [16], it also proposed three-dimensional time and replacement of the Lorentz transformation with vector Lorentz transformations. References [17–19] discuss the time dilation phenomenon on the basis of the special theory of ether. Also, the paper [20] presents a method for parameterizing new Lorentz spacetime coordinates based on coupled parameters. The works [21–23] analyze the phenomenon of space contraction along *X*, *Y*, and *Z*-directions when the motion between frames takes place in the three dimensions of space. The author of article [24] presented an original derivation of Lorentz transformation equations in 3 + 3 dimensions of spacetime continuum.

The presentation of this study is arranged as follows.  Section 2 presents the mathematical derivation of the Lorentz transformation equations between inertial frames of reference moving in a 2D *XY*-plane, including the form of these transformations in 2 + 2 dimensions.  Section 3 focuses on the reformulation of the velocity addition formula while Section 4 encompasses the concept of four-vector in 2 + 2 dimensions, and Section 5 gives the mathematical interpretation for the transformation of momentum and energy.  Section 6 presents the 2D Lorentz transformations in hyperbolic form while Section 7 discusses the invariance of the D'Alembert operator in 2 + 2 dimensions of spacetime continuum. Concluding comments then follow in Section 8.

## 2. Lorentz Transformation Equations in 2 + 2 Dimensions

**Figure 1:**
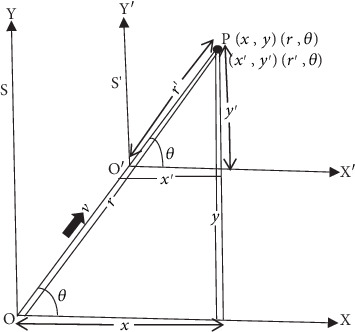
Inertial frames of reference moving in 2D *XY*-plane.

In Figure 1, we take a rectangular Cartesian frame *S* and another frame *S*′ moving with a uniform velocity *v* relative to the first frame, and the motion is assumed to be along the diagonal line OP in 2D *XY*-plane. Here, we view the relative velocity *v* as a parameter measuring the departure of current frame *S*′ from the rest frame *S* in 2D space. Looking at Figure 1, we see that the spatial coordinate in the *Z*-directions satisfies *z* = *z*′ = 0, so we leave *Z*-coordinate out of our equations further on, especially to keep mathematical calculations simple. In Figure 1, the Cartesian coordinates of point *P* measured from frame *S* and *S*′ are denoted by (*x*, *y*, *z* = 0) and (*x*′, *y*′, *z*′ = 0), respectively. In the same way, the corresponding polar coordinates of a point in 2D *XY*-plane as measured from rest frame *S* and current frame *S*′ are represented by (*r*, *θ*) and (*r*′, *θ*), respectively. Here, the angle *θ* must be the same for observers from both *S* system and *S*′ system due to symmetric space contraction in the *X* and *Y*-directions. If the motion between frames of reference is in the two dimensions of space, then there is the simultaneous space contraction in the *X* and *Y*-directions by the same Lorentz factor which obviously keeps the angle *θ* same in both frames of reference. It is given in more detail in the article [23]. One can apply the trigonometry in Figure 1 to know the relation between Cartesian (*x*, *y*) and polar coordinate (*r*, *θ*) of a point *P* in frame *S* such that

(3) $$\begin{array}{c} x=r\cos\theta , \end{array}$$

(4) $$\begin{array}{c} y=r\sin\theta . \end{array}$$

On applying Pythagorean theorem in Figure 1, we may show that

(5) $$\begin{array}{c} r^{2}=x^{2}+y^{2}. \end{array}$$

In exactly the same way, one can apply the trigonometry in Figure 1 to know the relation between Cartesian (*x*′, *y*′) and polar coordinate (*r*′, *θ*) of a point *P* in frame *S*′ such that

(6) $$\begin{array}{c} x^{\prime }=r^{\prime }\cos\theta , \end{array}$$

(7) $$\begin{array}{c} y^{\prime }=r^{\prime }\sin\theta . \end{array}$$

On applying Pythagorean theorem in Figure 1, we may show that

(8) $$\begin{array}{c} {r^{\prime }}^{2}={x^{\prime }}^{2}+{y^{\prime }}^{2}. \end{array}$$

Suppose time is measured from the rest frame with variable *t* and from the moving frame with variable *t*′. Now, consider a beam of light emitted in *XY*-plane along diagonal line *OP* at the instant when the origins of *S* and *S*′ coincide at *t* = *t*′ = 0. When it reaches to point *P*, let observer at rest in frame *S* record this event at position (*x*, *y*, *z* = 0) at time *t*. Relative to frame *S* the light would appear to have travelled the path *OP* as shown in Figure 1 such that

(9) $$\begin{array}{c} \frac{OP}{t}=\frac{r}{t}=c,\\ r=ct. \end{array}$$

Having taken Equation (5) into account, we obtain

(10) $$\begin{array}{c} \sqrt{x^{2}+y^{2}}=ct,\\ x^{2}+y^{2}=c^{2}t^{2}. \end{array}$$

Let the observer at rest in frame *S*′ record the same event at position (*x*′, *y*′, *z*′ = 0) at time *t*′. Relative to frame *S*′ the light would appear to have travelled the path *O*′*P* as shown in Figure 1 such that

(11) $$\begin{array}{c} \frac{O^{\prime }P}{t^{\prime }}=\frac{r^{\prime }}{t^{\prime }}=c,\\ r^{\prime }=ct^{\prime }. \end{array}$$

Having taken Equation (8) into account, we obtain

(12) $$\begin{array}{c} \sqrt{{x^{\prime }}^{2}+{y^{\prime }}^{2}}=ct^{\prime },\\ {x^{\prime }}^{2}+{y^{\prime }}^{2}=c^{2}{t^{\prime }}^{2}. \end{array}$$

Subtracting Equation (12) from (10), one obtains

(13) $$\begin{array}{c} x^{2}+y^{2}-\left({x^{\prime }}^{2}+{y^{\prime }}^{2}\right)=c^{2}t^{2}-c^{2}{t^{\prime }}^{2},\\ x^{2}+y^{2}-c^{2}t^{2}={x^{\prime }}^{2}+{y^{\prime }}^{2}-c^{2}{t^{\prime }}^{2},\\ r^{2}-c^{2}t^{2}={r^{\prime }}^{2}-c^{2}{t^{\prime }}^{2}. \end{array}$$

Equation (13) represents the invariance of spacetime interval equation along the diagonal line *OP* as indicated in Figure 1. Now multiplying both sides of Equation (13) by cos^2^*θ* and taking Equations (3) and (6) into account, one obtains

(14) $$\begin{array}{c} r^{2}\cos^{2}\theta -c^{2}t^{2}\cos^{2}\theta ={r^{\prime }}^{2}\cos^{2}\theta -c^{2}{t^{\prime }}^{2}\cos^{2}\theta ,\\ x^{2}-c^{2}t^{2}\cos^{2}\theta ={x^{\prime }}^{2}-c^{2}{t^{\prime }}^{2}\cos^{2}\theta . \end{array}$$

Equation (14) represents the invariance of spacetime interval equation in the *X*-direction. In exactly the same way multiplying both sides of Equation (13) by sin^2^*θ* and taking Equations (4) and (7) into account, one obtains

(15) $$\begin{array}{c} r^{2}\sin^{2}\theta -c^{2}t^{2}\sin^{2}\theta ={r^{\prime }}^{2}\sin^{2}\theta -c^{2}{t^{\prime }}^{2}\sin^{2}\theta ,\\ y^{2}-c^{2}t^{2}\sin^{2}\theta ={y^{\prime }}^{2}-c^{2}{t^{\prime }}^{2}\sin^{2}\theta . \end{array}$$

Equation (15) represents the invariance of spacetime interval equation in the *Y*-direction. In accordance with Figure 1, we have

$$\begin{array}{cc} \; & O^{\prime }P=OP-OO^{\prime },\\ r^{\prime }=r-vt. \end{array}$$

This relation is called Gallian transformation. To make it valid in relativistic mechanics, it must be multiplied by Lorentz factor *γ* as follows:

(16) $$\begin{array}{c} r^{\prime }=\gamma \left(r-vt\right). \end{array}$$

Substituting the value of *t* from Equation (9) into (16), one obtains

(17) $$\begin{array}{c} r^{\prime }=\gamma \left(r-\frac{vr}{c}\right)=\gamma r\left(1-\frac{v}{c}\right). \end{array}$$

Similarly, in accordance with Figure 1, we have

$$\begin{array}{cc} \; & OP=O^{\prime }P+OO^{\prime },\\ r=r^{\prime }+vt^{\prime }. \end{array}$$

This relation is called Gallian transformation. To make it valid in relativistic mechanics, it must be multiplied by Lorentz factor *γ* as follows:

(18) $$\begin{array}{c} r=\gamma \left(r^{\prime }+vt^{\prime }\right). \end{array}$$

Substituting the value of *t*′ from Equation (11) into (18), one obtains

 $$\begin{array}{c} r=\gamma \left(r^{\prime }+\frac{vr^{\prime }}{c}\right)=\gamma r^{\prime }\left(1+\frac{v}{c}\right). \end{array}$$

Introducing the value of *r*′ from Equation (17), the foregoing equation can be written as

(19) $$\begin{array}{c} r=r\gamma^{2}\left(1-\frac{v}{c}\right)\left(1+\frac{v}{c}\right),\\ \gamma =\frac{1}{\sqrt{1-\left(v^{2}/c^{2}\right)}}. \end{array}$$

After substituting Equation (19) into Equations (16) and (18), we obtain

(20) $$\begin{array}{c} r^{\prime }=\frac{r-vt}{\sqrt{1-\left(v^{2}/c^{2}\right)}}, \end{array}$$

(21) $$\begin{array}{c} r=\frac{r^{\prime }+vt^{\prime }}{\sqrt{1-\left(v^{2}/c^{2}\right)}}. \end{array}$$

Transformations (20) and (21) give the Lorentz transformation equation in terms of resultant radius vectors *r* and *r*′ when frames of reference move in 2D *XY*-plane. Now another important task is to find the transformation equations in the *X* and *Y*-directions. Transformation equations in the *X*-direction can be achieved by substituting the value of *r*′ from Equation (6) into (20) as follows:

$$\begin{array}{cc} \; & \frac{x^{\prime }}{\cos\theta }=\frac{r-vt}{\sqrt{1-\left(v^{2}/c^{2}\right)}},\\ x^{\prime }=\frac{r\cos\theta -vt\cos\theta }{\sqrt{1-\left(v^{2}/c^{2}\right)}}. \end{array}$$

Having taken Equation (3) into account, we obtain

(22) $$\begin{array}{c} x^{\prime }=\frac{x-vt\cos\theta }{\sqrt{1-\left(v^{2}/c^{2}\right)}}. \end{array}$$

Also substituting the value of cos*θ* from Equation (3) into (22), one obtains

(23) $$\begin{array}{c} x^{\prime }=\frac{x-\left(vtx/r\right)}{\sqrt{1-\left(v^{2}/c^{2}\right)}},\\ x^{\prime }=\frac{x-\left(vtx/\sqrt{x^{2}+y^{2}}\right)}{\sqrt{1-\left(v^{2}/c^{2}\right)}}. \end{array}$$

Equations (22) and (23) give the direct Lorentz transformation in the *X*-direction when the motion between inertial frames takes place in 2D *XY*-plane. Similarly, transformation equations in the *Y*-direction can be achieved by substituting the value of *r*′ from Equation (7) into (20) as follows:

$$\begin{array}{cc} \; & \frac{y^{\prime }}{\sin\theta }=\frac{r-vt}{\sqrt{1-\left(v^{2}/c^{2}\right)}},\\ y^{\prime }=\frac{r\sin\theta -vt\sin\theta }{\sqrt{1-\left(v^{2}/c^{2}\right)}}. \end{array}$$

Having taken Equation (4) into account, we obtain

(24) $$\begin{array}{c} y^{\prime }=\frac{y-vt\sin\theta }{\sqrt{1-\left(v^{2}/c^{2}\right)}}. \end{array}$$

Also substituting the value of sin*θ* from Equation (4) into (24), one obtains

(25) $$\begin{array}{c} y^{\prime }=\frac{y-\left(vty/r\right)}{\sqrt{1-\left(v^{2}/c^{2}\right)}},\\ y^{\prime }=\frac{y-\left(vty/\sqrt{x^{2}+y^{2}}\right)}{\sqrt{1-\left(v^{2}/c^{2}\right)}}. \end{array}$$

Equations (24) and (25) give the direct Lorentz transformation in the *Y*-direction when the motion between inertial frames takes place in 2D *XY*-plane. Inverse transformation equation in the *X*-direction can be achieved by substituting the value of *r* from Equation (3) into (21) as follows:

$$\begin{array}{cc} \; & \frac{x}{\cos\theta }=\frac{r^{\prime }+vt^{\prime }}{\sqrt{1-\left(v^{2}/c^{2}\right)}},\\ x=\frac{r^{\prime }\cos\theta +vt^{\prime }\cos\theta }{\sqrt{1-\left(v^{2}/c^{2}\right)}}. \end{array}$$

Having taken Equation (6) into account, we obtain

 $$\begin{array}{c} x=\frac{x^{\prime }+vt^{\prime }\cos\theta }{\sqrt{1-\left(v^{2}/c^{2}\right)}}. \end{array}$$

Also substituting the value of cos*θ* from Equation (6), the foregoing equation can be written as

 $$\begin{array}{c} x=\frac{x^{\prime }+\left(vt^{\prime }x^{\prime }/r^{\prime }\right)}{\sqrt{1-\left(v^{2}/c^{2}\right)}}=\frac{x^{\prime }+\left(vt^{\prime }x^{\prime }/\sqrt{{x^{\prime }}^{2}+{y^{\prime }}^{2}}\right)}{\sqrt{1-\left(v^{2}/c^{2}\right)}}. \end{array}$$

This equation expresses the inverse Lorentz transformation in the *X*-direction when the motion between inertial frames takes place in 2D *XY*-plane. Similarly, inverse transformation equations in the *Y*-direction can be achieved by substituting the value of *r* from Equation (4) into (21) as follows:

$$\begin{array}{cc} \; & \frac{y}{\sin\theta }=\frac{r^{\prime }+vt^{\prime }}{\sqrt{1-\left(v^{2}/c^{2}\right)}},\\ y=\frac{r^{\prime }\sin\theta +vt^{\prime }\sin\theta }{\sqrt{1-\left(v^{2}/c^{2}\right)}}. \end{array}$$

Having taken Equation (7) into account, we obtain

 $$\begin{array}{c} y=\frac{y^{\prime }+vt^{\prime }\sin\theta }{\sqrt{1-\left(v^{2}/c^{2}\right)}}. \end{array}$$

Also substituting the value of sin*θ* from Equation (7), the foregoing equation can be written as

 $$\begin{array}{c} y=\frac{y^{\prime }+\left(vt^{\prime }y^{\prime }/r^{\prime }\right)}{\sqrt{1-\left(v^{2}/c^{2}\right)}}=\frac{y^{\prime }+\left(vt^{\prime }y^{\prime }/\sqrt{{x^{\prime }}^{2}+{y^{\prime }}^{2}}\right)}{\sqrt{1-\left(v^{2}/c^{2}\right)}}. \end{array}$$

This equation expresses the inverse Lorentz transformation in the *Y*-direction when the motion between inertial frames takes place in 2D *XY*-plane. In order to deduce the transformation equation of time, we substitute the value of *t* from Equation (9) into Equation (20) as follows:

 $$\begin{array}{c} r^{\prime }=\frac{r-vt}{\sqrt{1-\left(v^{2}/c^{2}\right)}}=\frac{r-\left(vr/c\right)}{\sqrt{1-\left(v^{2}/c^{2}\right)}}. \end{array}$$

Having taken Equations (9) and (11) into account, we obtain

(26) $$\begin{array}{c} {ct}^{\prime }=\frac{ct-\left(vr/c\right)}{\sqrt{1-\left(v^{2}/c^{2}\right)}},\\ t^{\prime }=\frac{t-\left(vr/c^{2}\right)}{\sqrt{1-\left(v^{2}/c^{2}\right)}}=\frac{t-\left(v\sqrt{x^{2}+y^{2}}/c^{2}\right)}{\sqrt{1-\left(v^{2}/c^{2}\right)}}. \end{array}$$

The corresponding inverse form of transformation (26) follows by replacing *v* by −*v* and changing primed and unprimed variables and it gets the form as follows:

(27) $$\begin{array}{c} t=\frac{t^{\prime }+\left(vr^{\prime }/c^{2}\right)}{\sqrt{1-\left(v^{2}/c^{2}\right)}}=\frac{t^{\prime }+\left(v\sqrt{{x^{\prime }}^{2}+{y^{\prime }}^{2}}/c^{2}\right)}{\sqrt{1-\left(v^{2}/c^{2}\right)}}. \end{array}$$

We have derived the form of Lorentz transformations, namely, Equations (23), (25), and (26), between inertial frames of reference moving in the 2D *XY*-plane. These equations have been further analyzed with different special cases of relative motion between the inertial frames in Table 1.

One of the most fascinating properties of Lorentz transformations is that the square of spacetime interval given by Equation (13) must be a Lorentz scalar, that is, Lorentz transformation equations preserve the square of spacetime interval:

 $$\begin{array}{c} x^{2}+y^{2}-c^{2}t^{2}={x^{\prime }}^{2}+{y^{\prime }}^{2}-c^{2}{t^{\prime }}^{2}. \end{array}$$

After substituting Equations (23), (25), and (26) into right hand side of the above equation, we obtain

 $$\begin{array}{c} {x^{\prime }}^{2}+{y^{\prime }}^{2}-c^{2}{t^{\prime }}^{2}={\left(\frac{x-\left(vtx/\sqrt{x^{2}+y^{2}}\right)}{\sqrt{1-\left(v^{2}/c^{2}\right)}}\right)}^{2}+{\left(\frac{y-\left(vty/\sqrt{x^{2}+y^{2}}\right)}{\sqrt{1-\left(v^{2}/c^{2}\right)}}\right)}^{2}-c^{2}{\left(\frac{t-\left(v\sqrt{x^{2}+y^{2}}/c^{2}\right)}{\sqrt{1-\left(v^{2}/c^{2}\right)}}\right)}^{2}=\frac{x^{2}+y^{2}-\left(2vt\left(x^{2}+y^{2}\right)/\sqrt{x^{2}+y^{2}}\right)+{\left(vt\right)}^{2}\left(\left(x^{2}+y^{2}\right)/\left(x^{2}+y^{2}\right)\right)-c^{2}t^{2}+2tv\sqrt{x^{2}+y^{2}}-\left(v^{2}\left(x^{2}+y^{2}\right)/c^{2}\right)}{1-\left(v^{2}/c^{2}\right)}=\frac{x^{2}+y^{2}+{\left(vt\right)}^{2}-c^{2}t^{2}-\left(v^{2}\left(x^{2}+y^{2}\right)/c^{2}\right)}{1-\left(v^{2}/c^{2}\right)}=\frac{\left(x^{2}+y^{2}\right)\left(1-\left(v^{2}/c^{2}\right)\right)-c^{2}t^{2}\left(1-\left(v^{2}/c^{2}\right)\right)}{1-\left(v^{2}/c^{2}\right)}=x^{2}+y^{2}-c^{2}t^{2}. \end{array}$$

Thus, we have clearly proved that *x*′^2^ + *y*′^2^ − *c*^2^*t*′^2^ = *x*^2^ + *y*^2^ − *c*^2^*t*^2^. Hence, our 2D transformation equations, namely, Equations (23), (25), and (26), preserve the form of the square of spacetime interval equation. Now our main task is to put these transformations into an appropriate matrix form. Here we will have two matrix equations of Lorentz transformations; one is for resultant space coordinates, namely, for *r* and *r*′, and another is for the components of resultant space coordinates, namely, for (*x*, *y*) and (*x*′, *y*′). In order to obtain the matrix form of resultant space coordinates *r* and *r*′, we consider the spacetime interval equation along the diagonal line from Equation (13):

$$\begin{array}{cc} \; & r^{2}-c^{2}t^{2}={r^{\prime }}^{2}-c^{2}{t^{\prime }}^{2},\\ r^{2}+{\left(\;ict\right)}^{2}={r^{\prime }}^{2}+{\left(\;ict^{\prime }\right)}^{2}. \end{array}$$

This invariance of spacetime interval equation indicates that *ict* must be the corresponding time coordinate of resultant space coordinate *r*. Thus, we have to write the transformation Equation (20) in terms of *r* and *ict* as follows:

(28) $$\begin{array}{c} r^{\prime }=\frac{r-vt}{\sqrt{1-\left(v^{2}/c^{2}\right)}}=\gamma \left(r-vt\right),\\ r^{\prime }=\gamma \left(r-\beta ct\right)=\gamma \left(r+i^{2}\beta ct\right),\\ r^{\prime }=\gamma \left(r+i\beta i\text{c}t\right). \end{array}$$

Here *β* = *v*/*c* and $\gamma =1/\sqrt{1-\beta^{2}}$. In the same way, we can write the transformation Equation (26) in terms of *r* and *ict* as follows:

(29) $$\begin{array}{c} t^{\prime }=\gamma \left(t-\frac{vr}{c^{2}}\right),\\ ict^{\prime }=\gamma \left(ict-i\beta r\right). \end{array}$$

Finally, we can package the Lorentz Transformations (28) and (29) into a 2 × 2 matrix form as follows:

(30) $$\begin{array}{c} \left[\begin{array}{c} r^{\prime }\\ {ict}^{\prime } \end{array}\right]=\left[\begin{array}{cc} \gamma & i\beta \gamma \\ -i\beta \gamma & \gamma \end{array}\right]\left[\begin{array}{c} r\\ ict \end{array}\right]. \end{array}$$

The corresponding inverse transformation follows by replacing *β* by −*β* and interchanging the primed and unprimed variables as follows:

(31) $$\begin{array}{c} \left[\begin{array}{c} r\\ ict \end{array}\right]=\left[\begin{array}{cc} \gamma & -i\beta \gamma \\ i\beta \gamma & \gamma \end{array}\right]\left[\begin{array}{c} r^{\prime }\\ {ict}^{\prime } \end{array}\right]. \end{array}$$

Transformations (30) and (31) express the matrix form for the Lorentz transformation of resultant spacetime coordinates. Now our main goal is to express the Lorentz transformations of the *X* and *Y*-directions into an appropriate matrix form. Based on Equation (3) and (4), the *X* and *Y*-components of resultant radius vector *r* in rest frame *S* are given by

(32) $$\begin{array}{c} z_{1}=x=r\cos\theta , \end{array}$$

(33) $$\begin{array}{c} z_{2}=y=r\sin\theta . \end{array}$$

Based on Equation (6) and (7), the *X* and *Y*-components of resultant radius vector *r*′ in moving frame *S*′ are given by

(34) $$\begin{array}{c} z_{1}^{\prime }=x^{\prime }=r^{\prime }\cos\theta , \end{array}$$

(35) $$\begin{array}{c} z_{2}^{\prime }=y^{\prime }=r^{\prime }\sin\theta . \end{array}$$

Up until now, we have obtained the *X* and *Y*-components of space coordinates *r* and *r*′. Now our task is to find the components of time coordinates *ict* and *ict*′. In Figure 1, the motion between frames and emitted pulse of light are allowed to propagate along the radial line *OP* in 2D *XY*-plane such that we can break the resultant velocity of light *c* into *X*-component *c*_*x*_ = *c*cos*θ* and *Y*-component *c*_*y*_ = *c*sin*θ*. With these components of resultant velocity of light *c*, the *X* and *Y*-components of time coordinate *ict* are *z*_3_ = *ict*cos*θ* and *z*_4_ = *ict*sin*θ*, respectively. Also, having taken Equation (14) into account, we obtain

(36) $$\begin{array}{c} x^{2}-c^{2}t^{2}\cos^{2}\theta ={x^{\prime }}^{2}-c^{2}{t^{\prime }}^{2}\cos^{2}\theta ,\\ \left\{\begin{array}{c} {\left(\;{\text{z}}_{1}\right)}^{2}+{\left(\;ict\cos\theta \right)}^{2}={\left(z_{1}^{\prime }\right)}^{2}+{\left(\;ict^{\prime }\cos\theta \right)}^{2}\\ {\left(\;z_{1}\right)}^{2}+{\left(\;z_{3}\right)}^{2}={\left(z_{1}^{\prime }\right)}^{2}+{\left(z_{3}^{\prime }\right)}^{2} \end{array}.\right. \end{array}$$

Equation (36) expresses the invariance of the spacetime interval equation in the *X*-direction when the motion between frames of reference takes place in 2D *XY*-plane. Looking a little more closely at Equation (36), it becomes evident that *z*_3_ = *ict*cos*θ* has to be the *X*-component of the time coordinate *ict* in exactly the same way as *z*_1_ = *x* = *r*cos*θ* being the *X*-component of the space coordinate *r*. Similarly, having taken Equation (15) into account, we obtain

(37) $$\begin{array}{c} y^{2}-c^{2}t^{2}\sin^{2}\theta ={y^{\prime }}^{2}-c^{2}{t^{\prime }}^{2}\sin^{2}\theta ,\\ \left\{\begin{array}{c} {\left(\;z_{2}\right)}^{2}+{\left(\;ict\sin\theta \right)}^{2}={\left(z_{2}^{\prime }\right)}^{2}+{\left(\;ict^{\prime }\sin\theta \right)}^{2}\\ {\left(\;z_{2}\right)}^{2}+{\left(\;z_{4}\right)}^{2}={\left(z_{2}^{\prime }\right)}^{2}+{\left(z_{4}^{\prime }\right)}^{2} \end{array}.\right. \end{array}$$

Equation (37) expresses the invariance of the spacetime interval equation in the *Y*-direction when the motion between frames of reference takes place in 2D *XY*-plane. Looking a little more closely at Equation (37), it becomes evident that *z*_4_ = *ict*sin*θ* has to be the *Y*-component of the time coordinate *ict* in exactly the same way as *z*_2_ = *y* = *r*sin*θ* being the *Y*-component of the space coordinate *r*. In view of Schemes (36) and (37), it then follows that the *X* and *Y*-components of time coordinate *ict* in frame *S* will be given by relations

(38) $$\begin{array}{c} z_{3}=ict\cos\theta , \end{array}$$

(39) $$\begin{array}{c} z_{4}=ict\sin\theta . \end{array}$$

Similarly, in view of Schemes (36) and (37), it then follows that the *X* and *Y*-components of time coordinate *ict*′  in frame *S*′ will be given by relations

(40) $$\begin{array}{c} z_{3}^{\prime }=ict^{\prime }\cos\theta , \end{array}$$

(41) $$\begin{array}{c} z_{4}^{\prime }=ict^{\prime }\sin\theta . \end{array}$$

Finally, we have determined the *X* and *Y*-components of resultant spacetime coordinates, namely, of *r* and *ict*, as displayed in Equations (32), (33), (38), and (39). Now we wish to express the Lorentz transformation Equations (22), (24), and (26) in terms of (*z*_1_, *z*_2_, *z*_3_, *z*_4_). For this, consider the transformation equation of the *X*-direction from Equation (22) as follows:

$$\begin{array}{cc} \; & x^{\prime }=\frac{x-vt\cos\theta }{\sqrt{1-\left(v^{2}/c^{2}\right)}}=\gamma \left(x-vt\cos\theta \right),\\ x^{\prime }=\gamma \left(x-\beta ct\cos\theta \right)=\gamma \left(x+i^{2}\beta ct\cos\theta \right),\\ x^{\prime }=\gamma \left(x+i\beta ict\cos\theta \right). \end{array}$$

Having taken Equations (32), (34), and (38) into account, we obtain

(42) $$\begin{array}{c} z_{1}^{\prime }=\gamma \left(z_{1}+i\beta z_{3}\right). \end{array}$$

Similarly, consider the transformation equation of the *Y*-direction from Equation (24) as follows:

$$\begin{array}{cc} \; & y^{\prime }=\frac{y-vt\sin\theta }{\sqrt{1-\left(v^{2}/c^{2}\right)}}=\gamma \left(y-vt\sin\theta \right),\\ y^{\prime }=\gamma \left(y-\beta ct\sin\theta \right)=\gamma \left(\text{y}+i^{2}\beta ct\sin\theta \right),\\ y^{\prime }=\gamma \left(y+i\beta ict\sin\theta \right). \end{array}$$

Having taken Equations (33), (35), and (39) into account, we obtain

(43) $$\begin{array}{c} z_{2}^{\prime }=\gamma \left(z_{2}+i\beta z_{4}\right). \end{array}$$

Rearranging Equation (26), we obtain

(44) $$\begin{array}{c} t^{\prime }=\gamma \left(t-\frac{vr}{c^{2}}\right),\\ ict^{\prime }=\gamma \left(ict-i\beta r\right). \end{array}$$

Multiplying both sides of Equation (44) by cos*θ*, we obtain

 $$\begin{array}{c} ict^{\prime }\cos\theta =\gamma \left(ict\cos\theta -i\beta r\cos\theta \right). \end{array}$$

Having taken Equations (32), (38), and (40) into account, we obtain

(45) $$\begin{array}{c} z_{3}^{\prime }=\gamma \left(z_{3}-i\beta z_{1}\right). \end{array}$$

Similarly, multiplying both sides of Equation (44) by sin*θ*, we obtain

 $$\begin{array}{c} ict^{\prime }\sin\theta =\gamma \left(ict\sin\theta -i\beta r\sin\theta \right). \end{array}$$

Having taken Equations (33), (39), and (41) into account, we obtain

(46) $$\begin{array}{c} z_{4}^{\prime }=\gamma \left(z_{4}-i\beta z_{2}\right). \end{array}$$

It should be remembered that Equations (42) and (43) describe the transformations for the *X* and *Y*-components of space coordinate *r* while Equations (45) and (46) describe the transformations for the corresponding components of time coordinate *ict*. Finally, we can package the Lorentz transformation Equations (42), (43), (45), and (46) into a 4 × 4 matrix form as follows:

(47) $$\begin{array}{c} \left[\begin{array}{c} z_{1}^{\prime }\\ z_{2}^{\prime }\\ \begin{array}{c} z_{3}^{\prime }\\ z_{4}^{\prime } \end{array} \end{array}\right]=\left[\begin{array}{cccc} \gamma & 0 & i\beta \gamma & 0\\ 0 & \gamma & 0 & i\beta \gamma \\ -i\beta \gamma & 0 & \gamma & 0\\ 0 & -i\beta \gamma & 0 & \gamma \end{array}\right]\left[\begin{array}{c} z_{1}\\ z_{2}\\ z_{3}\\ z_{4} \end{array}\right]. \end{array}$$

This is the matrix equation of Lorentz transformation equations between inertial frames of reference moving in 2D *XY*-plane. The corresponding inverse transformation follows by replacing *β* by −*β* and interchanging the primed and unprimed variables as follows:

(48) $$\begin{array}{c} \left[\begin{array}{c} z_{1}\\ z_{2}\\ z_{3}\\ z_{4} \end{array}\right]=\left[\begin{array}{cccc} \gamma & 0 & -i\beta \gamma & 0\\ 0 & \gamma & 0 & -i\beta \gamma \\ i\beta \gamma & 0 & \gamma & 0\\ 0 & i\beta \gamma & 0 & \gamma \end{array}\right]\left[\begin{array}{c} z_{1}^{\prime }\\ z_{2}^{\prime }\\ \begin{array}{c} z_{3}^{\prime }\\ z_{4}^{\prime } \end{array} \end{array}\right]. \end{array}$$

Also, adding Equations (36) and (37), we obtain

 $$\begin{array}{c} {\left(\;z_{1}\right)}^{2}+{\left(\;z_{2}\right)}^{2}+{\left(\;z_{3}\right)}^{2}+{\left(\;z_{4}\right)}^{2}={\left(z_{1}^{\prime }\right)}^{2}+{\left(z_{2}^{\prime }\right)}^{2}+{\left(z_{3}^{\prime }\right)}^{2}+{\left(z_{4}^{\prime }\right)}^{2}. \end{array}$$

This equation represents the invariance of spacetime interval equation in 2 + 2 dimensions.

## 3. Lorentz Transformations of Physical Quantities

In the last section, we have presented the form of the Lorentz transformations between inertial frames moving in the 2D *XY*-plane. As the motion between frames is constrained in 2D *XY*-plane, we take *z*′ = *z* = 0 for convenience so that (*x*, *y*) and (*x*′, *y*′) are the variables of our interest. Now our task is to find the Lorentz velocity transformation equations for the particle moving in 2D *XY*-plane. Let us again consider two inertial systems of reference *S* and *S*′, the velocity of the second with respect to the first being *v*; their spacetime coordinates are connected by Formulas (22), (24), and (26). Then, the motion of an arbitrary point (which may be a material particle or merely a geometrical position) will be described by the function *r*(*t*) in system *S* and function *r*′(*t*′) in system *S*′. The instantaneous components of total velocity *u* of the moving material particle as measured in frame *S* will be given by the relations

(49) $$\begin{array}{c} u_{x}=\frac{dx}{dt},u_{y}=\frac{dy}{dt},u_{z}=0. \end{array}$$

Having taken Equations (3) and (4) into account, we obtain

(50) $$\begin{array}{c} u_{x}=\frac{dr}{dt}\cos\theta =u\cos\theta , \end{array}$$

(51) $$\begin{array}{c} u_{y}=\frac{dr}{dt}\sin\theta =u\sin\theta . \end{array}$$

And the total resultant velocity of the moving particle as measured in frame *S* will be given by the relation

(52) $$\begin{array}{c} u=\frac{dr}{dt}. \end{array}$$

If *m*_0_ be the rest mass of the particle, then its relativistic mass measured in frame *S* is given by

 $$\begin{array}{c} m=\frac{m_{0}}{\sqrt{1-\left(u^{2}/c^{2}\right)}}. \end{array}$$

Thus, the *X* and *Y*-components of total linear momentum in frame *S* are defined by the equations

(53) $$\begin{array}{c} p_{1}=mu_{x}=mu\cos\theta , \end{array}$$

(54) $$\begin{array}{c} p_{2}=p\sin\theta . \end{array}$$

And the total resultant momentum of the moving particle as measured in frame *S* will be given by the relation

(55) $$\begin{array}{c} p=mu. \end{array}$$

Similarly, the instantaneous components of total velocity *u*′ of the moving point as measured in frame *S*′ will be given by the relations

(56) $$\begin{array}{c} u_{x}^{\prime }=\frac{dx^{\prime }}{dt^{\prime }},u_{y}^{\prime }=\frac{dy^{\prime }}{dt^{\prime }},u_{z}^{\prime }=0. \end{array}$$

Having taken Equations (6) and (7) into account, we obtain

(57) $$\begin{array}{c} u_{x}^{\prime }=\frac{dr^{\prime }}{dt^{\prime }}\;\cos\theta =u^{\prime }\cos\theta , \end{array}$$

(58) $$\begin{array}{c} u_{y}^{\prime }=\frac{dr^{\prime }}{dt^{\prime }}\;\sin\theta =u^{\prime }\sin\theta . \end{array}$$

And the total resultant velocity of the moving particle as measured in frame *S*′ will be given by the relation

(59) $$\begin{array}{c} u^{\prime }=\frac{dr^{\prime }}{dt^{\prime }}. \end{array}$$

If *m*_0_ be the rest mass of the particle, then its relativistic mass measured in frame *S*′ is given by

 $$\begin{array}{c} m^{\prime }=\frac{m_{0}}{\sqrt{1-\left({u^{\prime }}^{2}/c^{2}\right)}}. \end{array}$$

Thus, the *X* and *Y*-components of total linear momentum in frame *S*′ are defined by the equations

(60) $$\begin{array}{c} p_{1}^{\prime }=m^{\prime }u_{x}^{\prime }=m^{\prime }u^{\prime }\cos\theta , \end{array}$$

(61) $$\begin{array}{c} p_{2}^{\prime }=p^{\prime }\sin\theta . \end{array}$$

And the total resultant momentum of the moving particle as measured in frame *S*′ will be given by the relation

(62) $$\begin{array}{c} p^{\prime }=m^{\prime }u^{\prime }. \end{array}$$

Consider (*x*, *y*) be the position of the particle at any instant of time *t* as measured from frame *S*, and the same particle is measured to be at the position (*x*′, *y*′) at time *t*′ from frame *S*′. Based on Transformations (22), (24), and (26), we write the following relations:

(63) $$\begin{array}{c} x^{\prime }=\gamma \left(x-vt\cos\theta \right), \end{array}$$

(64) $$\begin{array}{c} y^{\prime }=\gamma \left(y-vt\sin\theta \right), \end{array}$$

(65) $$\begin{array}{c} t^{\prime }=\gamma \left(t-\frac{vr}{c^{2}}\right). \end{array}$$

Let this particle be measured to be at the point (*x* + *dx*, *y* + *dy*) at a time *t* + *dt* from frame *S* and the same particle is measured to be at the point (*x*′ + *dx*′, *y*′ + *dy*′) at time *t*′ + *dt*′ from frame *S*′. Based on Transformations (22), (24), and (26), we write the following relations:

(66) $$\begin{array}{c} x^{\prime }+{dx}^{\prime }=\gamma \left[x+dx-v\left(t+dt\right)\cos\theta \right], \end{array}$$

(67) $$\begin{array}{c} y^{\prime }+{dy}^{\prime }=\gamma \left[y+dy-v\left(t+dt\right)\sin\theta \right], \end{array}$$

(68) $$\begin{array}{c} t^{\prime }+{dt}^{\prime }=\gamma \left[t+dt-\frac{v\left(r+dr\right)}{c^{2}}\right]. \end{array}$$

Subtracting Equation (63) from (66), one obtains

(69) $$\begin{array}{c} dx^{\prime }=\gamma \left(dx-v\cos\theta dt\right)=\gamma \left(\frac{dx}{dt}-v\cos\theta \right)dt,\\ dx^{\prime }=\gamma \left(u_{x}-v\cos\theta \right)dt. \end{array}$$

Subtracting Equation (64) from (67), one obtains

(70) $$\begin{array}{c} dy^{\prime }=\gamma \left(dy-v\sin\theta dt\right)=\gamma \left(\frac{dy}{dt}-v\sin\theta \right)dt,\\ dy^{\prime }=\gamma \left(u_{y}-v\sin\theta \right)dt. \end{array}$$

Subtracting Equation (65) from (68), one obtains

(71) $$\begin{array}{c} dt^{\prime }=\gamma \left(dt-\frac{v}{c^{2}}dr\right)=\gamma \left(1-\frac{v}{c^{2}}\frac{dr}{dt}\right)dt,\\ dt^{\prime }=\gamma \left(1-\frac{uv}{c^{2}}\right)dt. \end{array}$$

We introduce the differential Equations (69), (70), and (71) into (56). We obtain

(72) $$\begin{array}{c} u_{x}^{\prime }=\frac{u_{x}-v\cos\theta }{1-\left(uv/c^{2}\right)}, \end{array}$$

(73) $$\begin{array}{c} u_{y}^{\prime }=\frac{u_{y}-v\sin\theta }{1-\left(uv/c^{2}\right)}. \end{array}$$

Also, the differential form of space coordinates in terms of radius vector *r* and *r*′ can be written from Equation (20) as follows:

(74) $$\begin{array}{c} dr^{\prime }=\gamma \left(\frac{dr}{dt}-v\right)dt,\\ dr^{\prime }=\gamma \left(u-v\right)dt. \end{array}$$

We introduce the differential Equations (74) and (71) into (59). We obtain

(75) $$\begin{array}{c} u^{\prime }=\frac{u-v}{1-\left(uv/c^{2}\right)}. \end{array}$$

Transformation (75) expresses the transformation of total resultant velocity while Transformations (72) and (73) express the transformations of *X* and *Y*-components of velocity. It should be noted that these transformations are applicable when frames of reference move in a 2D *XY*-plane. The corresponding transformation follows by replacing *v* by −*v* and interchanging the primed and unprimed variables. The result is

(76) $$\begin{array}{c} u_{x}=\frac{u_{x}^{\prime }+v\cos\theta }{1+\left(u^{\prime }v/c^{2}\right)}, \end{array}$$

(77) $$\begin{array}{c} u_{y}=\frac{u_{y}^{\prime }+v\sin\theta }{1+\left(u^{\prime }v/c^{2}\right)}, \end{array}$$

(78) $$\begin{array}{c} u=\frac{u^{\prime }+v}{1+\left(u^{\prime }v/c^{2}\right)}. \end{array}$$

## 4. Concept of Four-Vector in 2 + 2 Dimensions

In ordinary four-dimensional Minkowski space, the three space coordinates (*x*, *y*, *z*) and one time coordinate *ict* give the notion of four-vector, which can be described from the invariance of space–time interval equation of one-dimensional Lorentz transformations. In this work, we have derived the 2D Lorentz transformation by extending the motion between inertial frames in 2D *XY*-plane. As a result of this extension, we have two time coordinates as described by the invariance of spacetime interval Equations (36) and (37). Hence, we have four space–time coordinates (*z*_1_, *z*_2_, *z*_3_, *z*_4_) in 2 + 2 dimensions out of which the first two represent the space coordinates (*x*, *y*) and last two represent the time coordinates. Thus, we need to analyze the coordinates (*z*_1_, *z*_2_, *z*_3_, *z*_4_) under our derived 2D transformations in exactly the same way as we had analyzed four-vector under ordinary one-dimensional Lorentz transformations. It should be always noted that the first two quantities represent the space part and the last two quantities represent the time part in our 2 + 2 dimensions, while in 3 + 1 dimensions the first three quantities represent the space part and the last one represents the time part. Based on the concept of four-vector, the four-velocity in frame *S*′ is defined as follows:

(79) $$\begin{array}{c} w_{i}^{\prime }=\frac{{dz}_{i}^{\prime }}{d\tau }=\frac{dz_{i}^{\prime }}{dt^{\prime }}\frac{dt^{\prime }}{d\tau },\\ w_{i}^{\prime }=\frac{dz_{i}^{\prime }}{dt^{\prime }\sqrt{1-\left({u^{\prime }}^{2}/c^{2}\right)}}. \end{array}$$

Here *τ* is the proper time, *t*′ is the relativistic time measured from moving frame *S*′, and *z*_*i*_′ is the position four-vector measured from the moving frame *S*′. In the same way, the expression of four-velocity of the same particle as seen from rest frame *S* is given by

(80) $$\begin{array}{c} w_{i}=\frac{{dz}_{i}}{dt\sqrt{1-\left(u^{2}/c^{2}\right)}}. \end{array}$$

Here *t* is the relativistic time measured from rest frame *S* and *z*_*i*_ is the position four-vector measured from rest frame *S*. Using Equations (32), (33), (38), and (39) into (80), we obtain four-velocity in our 2 + 2 dimensions as follows:

$$\begin{array}{cc} \; & w_{1}=\frac{dz_{1}}{dt\sqrt{1-\left(u^{2}/c^{2}\right)}}=\frac{dr}{dt}\frac{\cos\theta }{\sqrt{1-\left(u^{2}/c^{2}\right)}}=\frac{u\cos\theta }{\sqrt{1-\left(u^{2}/c^{2}\right)}},\\ w_{2}=\frac{dz_{2}}{dt\sqrt{1-\left(u^{2}/c^{2}\right)}}=\frac{dr}{dt}\frac{\sin\theta }{\sqrt{1-\left(u^{2}/c^{2}\right)}}=\frac{u\sin\theta }{\sqrt{1-\left(u^{2}/c^{2}\right)}},\\ w_{3}=\frac{dz_{3}}{dt\sqrt{1-\left(u^{2}/c^{2}\right)}}=\frac{dt}{dt}\frac{ic\cos\theta }{\sqrt{1-\left(u^{2}/c^{2}\right)}}=\frac{ic\cos\theta }{\sqrt{1-\left(u^{2}/c^{2}\right)}},\\ w_{4}=\frac{dz_{4}}{dt\sqrt{1-\left(u^{2}/c^{2}\right)}}=\frac{dt}{dt}\frac{ic\sin\theta }{\sqrt{1-\left(u^{2}/c^{2}\right)}}=\frac{ic\sin\theta }{\sqrt{1-\left(u^{2}/c^{2}\right)}}. \end{array}$$

Based on the notion of four-vector, we can define the four-momentum in rest frame S as follows.

 $$\begin{array}{c} p_{i}=m_{0}w_{i}, \end{array}$$

By substituting the value of (*w*_1_, *w*_2_, *w*_3_, *w*_4_), we obtain four-momentum in our 2 + 2 dimensions as follows:

$$\begin{array}{cc} \; & p_{1}=m_{0}w_{1}=\frac{m_{0}}{\sqrt{1-\left(u^{2}/c^{2}\right)}}u\cos\theta =mu\cos\theta =p\cos\theta ,\\ p_{2}=m_{0}w_{2}=\frac{m_{0}}{\sqrt{1-\left(u^{2}/c^{2}\right)}}u\sin\theta =mu\sin\theta =p\sin\theta ,\\ p_{3}=m_{0}w_{3}=\frac{m_{0}}{\sqrt{1-\left(u^{2}/c^{2}\right)}}ic\cos\theta =imc\cos\theta =\frac{iE}{c}\cos\theta ,\\ p_{4}=m_{0}w_{4}=\frac{m_{0}}{\sqrt{1-\left(u^{2}/c^{2}\right)}}ic\sin\theta =imc\sin\theta =\frac{iE}{c}\sin\theta . \end{array}$$

Thus, four-momentum in our 2 + 2 dimensions can be written as follows:

(81) $$\begin{array}{c} \left\{\begin{array}{cc} p_{1}=p\cos\theta , & p_{2}=p\sin\theta ,\\ p_{3}=\frac{iE}{c}\cos\theta , & p_{4}=\frac{iE}{c}\sin\theta . \end{array}\right. \end{array}$$

This is the expression of four-momentum in rest frame *S* with respect to which particle is moving with the velocity *u* and one can repeat exactly the same mathematical calculation in frame *S*′ with respect to which the same particle is moving with the velocity *u*′. We use Equations (34), (35), (40), and (41) into (79) to obtain four-velocity in moving frame *S*′ as follows:

$$\begin{array}{cc} \; & w_{1}^{\prime }=\frac{dz_{1}^{\prime }}{dt^{\prime }\sqrt{1-\left({u^{\prime }}^{2}/c^{2}\right)}}=\frac{dr^{\prime }}{dt^{\prime }}\frac{\cos\theta }{\sqrt{1-\left({u^{\prime }}^{2}/c^{2}\right)}}=\frac{u^{\prime }\cos\theta }{\sqrt{1-\left({u^{\prime }}^{2}/c^{2}\right)}},\\ w_{2}^{\prime }=\frac{dz_{2}^{\prime }}{dt^{\prime }\sqrt{1-\left({u^{\prime }}^{2}/c^{2}\right)}}=\frac{dr^{\prime }}{dt^{\prime }}\frac{\sin\theta }{\sqrt{1-\left({u^{\prime }}^{2}/c^{2}\right)}}=\frac{u^{\prime }\sin\theta }{\sqrt{1-\left({u^{\prime }}^{2}/c^{2}\right)}},\\ w_{3}^{\prime }=\frac{dz_{3}^{\prime }}{dt^{\prime }\sqrt{1-\left({u^{\prime }}^{2}/c^{2}\right)}}=\frac{dt^{\prime }}{dt^{\prime }}\frac{ic\cos\theta }{\sqrt{1-\left({u^{\prime }}^{2}/c^{2}\right)}}=\frac{ic\cos\theta }{\sqrt{1-\left({u^{\prime }}^{2}/c^{2}\right)}},\\ w_{4}^{\prime }=\frac{dz_{4}^{\prime }}{dt^{\prime }\sqrt{1-\left({u^{\prime }}^{2}/c^{2}\right)}}=\frac{dt^{\prime }}{dt^{\prime }}\frac{ic\sin\theta }{\sqrt{1-\left({u^{\prime }}^{2}/c^{2}\right)}}=\frac{ic\sin\theta }{\sqrt{1-\left({u^{\prime }}^{2}/c^{2}\right)}}. \end{array}$$

Based on the notion of four-vector, we can define the four-momentum in moving frame *S*′ as follows:

 $$\begin{array}{c} p_{i}=m_{0}w_{i}^{\prime }. \end{array}$$

By substituting the value of (*w*_1_′, *w*_2_′, *w*_3_′, *w*_4_′), we obtain four-momentum in our 2 + 2 dimensions as follows:

$$\begin{array}{cc} \; & p_{1}^{\prime }=m_{0}w_{1}^{\prime }=\frac{m_{0}}{\sqrt{1-\left({u^{\prime }}^{2}/c^{2}\right)}}u^{\prime }\cos\theta =m^{\prime }u^{\prime }\cos\theta =p^{\prime }\cos\theta ,\\ p_{2}^{\prime }=m_{0}w_{2}^{\prime }=\frac{m_{0}}{\sqrt{1-\left({u^{\prime }}^{2}/c^{2}\right)}}u^{\prime }\sin\theta =m^{\prime }u^{\prime }\sin\theta =p^{\prime }\sin\theta ,\\ p_{3}^{\prime }=m_{0}w_{3}^{\prime }=\frac{m_{0}}{\sqrt{1-\left({u^{\prime }}^{2}/c^{2}\right)}}ic\cos\theta =im^{\prime }c\cos\theta =\frac{iE^{\prime }}{c}\cos\theta ,\\ p_{4}^{\prime }=m_{0}w_{4}^{\prime }=\frac{m_{0}}{\sqrt{1-\left({u^{\prime }}^{2}/c^{2}\right)}}ic\sin\theta =im^{\prime }c\sin\theta =\frac{iE^{\prime }}{c}\sin\theta . \end{array}$$

Thus, four-momentum in our 2 + 2 dimensions as measured from moving frame *S*′ can be written as follows:

(82) $$\begin{array}{c} \left\{\begin{array}{cc} p_{1}^{\prime }=p^{\prime }\cos\theta , & p_{2}^{\prime }=p^{\prime }\sin\theta ,\\ p_{3}^{\prime }=\frac{iE^{\prime }}{c}\cos\theta , & p_{4}^{\prime }=\frac{iE^{\prime }}{c}\sin\theta . \end{array}\right. \end{array}$$

A quantity is regarded as a four-vector if it abides the same transformation as in (47) under a Lorentz boost. In view of Equation (47), the transformation of four-momentum in our 2 + 2 dimensions can be described as follows:

 $$\begin{array}{c} \left[\begin{array}{c} p_{2}^{\prime }\\ \begin{array}{c} p_{4}^{\prime } \end{array} \end{array}\right]=\left[\begin{array}{cccc} 0 & i\beta \gamma & 0\\ 0 & \gamma & 0 & i\beta \gamma \\ -i\beta \gamma & 0 & \gamma & 0\\ 0 & -i\beta \gamma & 0 & \gamma \end{array}\right]\left[\begin{array}{c} p_{2}\\ p_{3}\\ p_{4} \end{array}\right]. \end{array}$$

The solution of this matrix equation leads to the following relations:

(83) $$\begin{array}{c} \left\{\begin{array}{c} p_{1}^{\prime }=\gamma \left(p_{1}+i\beta p_{3}\right)\\ p_{2}^{\prime }=\gamma \left(p_{2}+i\beta p_{4}\right)\\ p_{3}^{\prime }=\gamma \left(p_{3}-i\beta p_{1}\right)\\ p_{4}^{\prime }=\gamma \left(p_{4}-i\beta p_{2}\right) \end{array}\right.. \end{array}$$

Most importantly, square of Minkowski length of a four-momentum must be invariant under a Lorentz transformation, that is,

(84) $$\begin{array}{c} {\left(p_{1}\right)}^{2}+{\left(p_{2}\right)}^{2}+{\left(p_{3}\right)}^{2}+{\left(p_{4}\right)}^{2}={\left(p_{1}^{\prime }\right)}^{2}+{\left(p_{2}^{\prime }\right)}^{2}+{\left(p_{3}^{\prime }\right)}^{2}+{\left(p_{4}^{\prime }\right)}^{2}. \end{array}$$

We introduce Equation (81) into left hand side of Equation (84). We obtain

(85) $$\begin{array}{c} {\left(p_{1}\right)}^{2}+{\left(p_{2}\right)}^{2}+{\left(p_{3}\right)}^{2}+{\left(p_{4}\right)}^{2}={\left(p\cos\theta \right)}^{2}+{\left(p\sin\theta \right)}^{2}+{\left(\frac{iE}{c}\cos\theta \right)}^{2}+{\left(\frac{iE}{c}\sin\theta \right)}^{2},\\ {\left(p_{1}\right)}^{2}+{\left(p_{2}\right)}^{2}+{\left(p_{3}\right)}^{2}+{\left(p_{4}\right)}^{2}=p^{2}\left(\cos^{2}\theta +\sin^{2}\theta \right)-\frac{E^{2}}{c^{2}}\left(\cos^{2}\theta +\sin^{2}\theta \right),\\ {\left(p_{1}\right)}^{2}+{\left(p_{2}\right)}^{2}+{\left(p_{3}\right)}^{2}+{\left(p_{4}\right)}^{2}=p^{2}-\frac{E^{2}}{c^{2}},\\ {\left(p_{1}\right)}^{2}+{\left(p_{2}\right)}^{2}+{\left(p_{3}\right)}^{2}+{\left(p_{4}\right)}^{2}=p^{2}-\frac{E^{2}}{c^{2}}. \end{array}$$

In the same way, we introduce Equation (82) into right hand side of Equation (84). We obtain

(86) $$\begin{array}{c} {\left(p_{1}^{\prime }\right)}^{2}+{\left(p_{2}^{\prime }\right)}^{2}+{\left(p_{3}^{\prime }\right)}^{2}+{\left(p_{4}^{\prime }\right)}^{2}={\left(p^{\prime }\cos\theta \right)}^{2}+{\left(p^{\prime }\sin\theta \right)}^{2}+{\left(\frac{iE^{\prime }}{c}\cos\theta \right)}^{2}+{\left(\frac{iE^{\prime }}{c}\sin\theta \right)}^{2},\\ {\left(p_{1}^{\prime }\right)}^{2}+{\left(p_{2}^{\prime }\right)}^{2}+{\left(p_{3}^{\prime }\right)}^{2}+{\left(p_{4}^{\prime }\right)}^{2}={p^{\prime }}^{2}\left(\cos^{2}\theta +\sin^{2}\theta \right)-\frac{{E^{\prime }}^{2}}{c^{2}}\left(\cos^{2}\theta +\sin^{2}\theta \right),\\ {\left(p_{1}^{\prime }\right)}^{2}+{\left(p_{2}^{\prime }\right)}^{2}+{\left(p_{3}^{\prime }\right)}^{2}+{\left(p_{4}^{\prime }\right)}^{2}={p^{\prime }}^{2}-\frac{{E^{\prime }}^{2}}{c^{2}},\\ {\left(p_{1}^{\prime }\right)}^{2}+{\left(p_{2}^{\prime }\right)}^{2}+{\left(p_{3}^{\prime }\right)}^{2}+{\left(p_{4}^{\prime }\right)}^{2}={p^{\prime }}^{2}-\frac{{E^{\prime }}^{2}}{c^{2}}. \end{array}$$

We introduce Equations (85) and (86) into (84). We obtain

(87) $$\begin{array}{c} \left\{\begin{array}{c} p^{2}-\frac{E^{2}}{c^{2}}={p^{\prime }}^{2}-\frac{{E^{\prime }}^{2}}{c^{2}},\\ p^{2}+{\left(\frac{iE}{c}\right)}^{2}={p^{\prime }}^{2}+{\left(\frac{iE^{\prime }}{c}\right)}^{2}. \end{array}\right. \end{array}$$

Equations (84) and (87) are the same equations and they represent the Lorentz invariant energy–momentum relations. In other words, Equation (84) gives the invariance of energy–momentum relations in terms of the components of momentum, that is, in terms of four-momentum while Equation (87) gives the invariance of same physical quantity in terms of resultant momentum, that is, in terms of *p* and *iE*/*c*. On making the comparison of Equation (84) with (87), it can be inferred that (*p*_1_, *p*_2_) must be the *X* and *Y*-components of space part of momentum *p* in exactly the same way as (*z*_1_, *z*_2_) being the *X* and *Y*-components of resultant space coordinate *r* and (*p*_3_, *p*_4_) be the components of time part of momentum *iE*/*c* in exactly same way as (*z*_3_, *z*_4_) being the components of resultant time coordinate *ict*. In Equation (83), the *X* and *Y*-components of energy–momentum (*p*_1_, *p*_2_, *p*_3_, *p*_4_) transform in exactly the same way as the transformation of the *X* and *Y*-components of spacetime coordinates (*z*_1_, *z*_2_, *z*_3_, *z*_4_). Similarly, the resultant energy–momentum (*p*, *iE*/*c*) must be transformed in the same way as the transformation of the resultant spacetime coordinates (*r*, *ict*) as delineated in Equation (30). Thus, replacing *r* by *p* and *ict* by *iE*/*c* in Equation (30), we obtain the following transformation relations:

 $$\begin{array}{c} \left[\begin{array}{c} iE^{\prime }/c \end{array}\right]=\left[\begin{array}{cc} i\beta \gamma \\ -i\beta \gamma & \gamma \end{array}\right]\left[\begin{array}{c} \;iE/c \end{array}\right]. \end{array}$$

The solution of above matrix equation gives the following transformation relations between total linear momentum and energy:

(88) $$\begin{array}{c} \left\{\begin{array}{c} p^{\prime }=\frac{p-Ev/c^{2}}{\sqrt{1-\left(v^{2}/c^{2}\right)}}, \end{array}E^{\prime }=\frac{E-pv}{\sqrt{1-\left(v^{2}/c^{2}\right)}}\right.. \end{array}$$

These are the well-known Lorentz transformation formulas for the total linear momentum and energy.

## 5. Transformation of Energy and Momentum

With reference to Figure 1, we take the frame *S*′ to be moving along a diagonal line *OP* in 2D *XY*-plane at velocity *v* relative to frame *S*. Consider a particle of rest *m*_0_ moving at velocity *u* in frame *S* and hence at velocity *u*′ in frame *S*′. Based on Equation (75), we have

(89) $$\begin{array}{c} u^{\prime }=\frac{u-v}{1-\left(uv/c^{2}\right)},\\ 1-\frac{{u^{\prime }}^{2}}{c^{2}}=\frac{{\left(1-\left(uv/c^{2}\right)\right)}^{2}-{\left(u-v/c\right)}^{2}}{{\left(1-\left(uv/c^{2}\right)\right)}^{2}},\\ 1-\frac{{u^{\prime }}^{2}}{c^{2}}=\frac{{\left(1-\left(uv/c^{2}\right)\right)}^{2}-{\left(\left(u-v\right)/c\right)}^{2}}{{\left(1-\left(uv/c^{2}\right)\right)}^{2}}=\frac{1+\left(u^{2}v^{2}/c^{4}\right)-\left(u^{2}/c^{2}\right)-\left(v^{2}/c^{2}\right)}{{\left(1-\left(uv/c^{2}\right)\right)}^{2}},\\ 1-\frac{{u^{\prime }}^{2}}{c^{2}}=\frac{\left(1-\left(u^{2}/c^{2}\right)\right)-\left(v^{2}/c^{2}\right)\left(1-\left(u^{2}/c^{2}\right)\right)}{{\left(1-\left(uv/c^{2}\right)\right)}^{2}}=\frac{\left(1-\left(u^{2}/c^{2}\right)\right)\left(1-\left(v^{2}/c^{2}\right)\right)}{{\left(1-\left(uv/c^{2}\right)\right)}^{2}},\\ \sqrt{1-\left({u^{\prime }}^{2}/c^{2}\right)}=\frac{\sqrt{\left(1-\left(u^{2}/c^{2}\right)\right)\left(1-\left(v^{2}/c^{2}\right)\right)}}{1-\left(uv/c^{2}\right)}. \end{array}$$

The energy and momentum in frame *S* are given by the relations:

$$\begin{array}{cc} \; & E=mc^{2}=\frac{m_{0}c^{2}}{\sqrt{1-\left(u^{2}/c^{2}\right)}},\\ p=mu=\frac{m_{0}u}{\sqrt{1-\left(u^{2}/c^{2}\right)}}. \end{array}$$

The corresponding quantities in frame *S*′ are given by relations:

(90) $$\begin{array}{c} E^{\prime }=m^{\prime }c^{2}=\frac{m_{0}c^{2}}{\sqrt{1-\left({u^{\prime }}^{2}/c^{2}\right)}}, \end{array}$$

(91) $$\begin{array}{c} p^{\prime }=m^{\prime }u^{\prime }=\frac{m_{0}u^{\prime }}{\sqrt{1-\left({u^{\prime }}^{2}/c^{2}\right)}}. \end{array}$$

Substituting Equation (89) into (90), we obtain

(92) $$\begin{array}{c} E^{\prime }=\frac{m_{0}c^{2}}{\sqrt{1-\left({u^{\prime }}^{2}/c^{2}\right)}}=\frac{m_{0}c^{2}\left(1-\left(uv/c^{2}\right)\right)}{\sqrt{\left(1-\left(u^{2}/c^{2}\right)\right)\left(1-\left(v^{2}/c^{2}\right)\right)}},\\ E^{\prime }=\frac{mc^{2}\left(1-\left(uv/c^{2}\right)\right)}{\sqrt{1-\left(v^{2}/c^{2}\right)}}=\frac{mc^{2}-muv}{\sqrt{1-\left(v^{2}/c^{2}\right)}},\\ E^{\prime }=\frac{E-pv}{\sqrt{1-\left(v^{2}/c^{2}\right)}}. \end{array}$$

Substituting Equations (75) and (89) into (91), we obtain

(93) $$\begin{array}{c} p^{\prime }=\frac{m_{0}u^{\prime }}{\sqrt{1-\left({u^{\prime }}^{2}/c^{2}\right)}}=\frac{m_{0}\left(1-\left(uv/c^{2}\right)\right)u^{\prime }}{\sqrt{\left(1-\left(u^{2}/c^{2}\right)\right)\left(1-\left(v^{2}/c^{2}\right)\right)}},\\ p^{\prime }=\frac{m_{0}\left(1-\left(uv/c^{2}\right)\right)}{\sqrt{\left(1-\left(u^{2}/c^{2}\right)\right)\left(1-\left(v^{2}/c^{2}\right)\right)}}\times \frac{\left(u-v\right)}{\left(1-\left(uv/c^{2}\right)\right)},\\ p^{\prime }=\frac{m_{0}\left(u-v\right)}{\sqrt{\left(1-\left(u^{2}/c^{2}\right)\right)\left(1-\left(v^{2}/c^{2}\right)\right)}}=\frac{mu-mv}{\sqrt{1-\left(v^{2}/c^{2}\right)}},\\ p^{\prime }=\frac{p-\left(Ev/c^{2}\right)}{\sqrt{1-\left(v^{2}/c^{2}\right)}}. \end{array}$$

Transformation Equations (93) and (92) of total resultant momentum and energy are exactly the same transformations as derived in Equation (88). In the preceding section, we had derived Transformation (88) with the direct use of the matrix equation of 2D Lorentz transformation while we have again exactly derived the same transformation with the use of 2D Lorentz transformation as given in Equations (92) and (93). Now another goal is to find the transformation equations for *X* and *Y*-components of total momentum. The *X*-component of resultant momentum in frame *S*′ can be written from Equation (60) as follows:

 $$\begin{array}{c} p_{1}^{\prime }=m^{\prime }u_{x}^{\prime }=\frac{m_{0}u_{x}^{\prime }}{\sqrt{1-\left({u^{\prime }}^{2}/c^{2}\right)}}. \end{array}$$

Having taken Equations (72) and (89) into account, we obtain

(94) $$\begin{array}{c} p_{1}^{\prime }=\frac{m_{0}\left(1-\left(uv/c^{2}\right)\right)u_{x}^{\prime }}{\sqrt{\left(1-\left(u^{2}/c^{2}\right)\right)\left(1-\left(v^{2}/c^{2}\right)\right)}},\\ p_{1}^{\prime }=\frac{m_{0}\left(1-\left(uv/c^{2}\right)\right)}{\sqrt{\left(1-\left(u^{2}/c^{2}\right)\right)\left(1-\left(v^{2}/c^{2}\right)\right)}}\times \frac{\left(u_{x}-v\cos\theta \right)}{\left(1-\left(uv/c^{2}\right)\right)},\\ p_{1}^{\prime }=\frac{m_{0}\left(u_{x}-v\cos\theta \right)}{\sqrt{\left(1-\left(u^{2}/c^{2}\right)\right)\left(1-\left(v^{2}/c^{2}\right)\right)}}=\frac{mu_{x}-mv\cos\theta }{\sqrt{1-\left(v^{2}/c^{2}\right)}},\\ p_{1}^{\prime }=\frac{p_{1}-\left(Ev\cos\theta /c^{2}\right)}{\sqrt{1-\left(v^{2}/c^{2}\right)}}. \end{array}$$

Similarly, the *Y*-component of resultant momentum in frame *S*′ can be written from Equation (61) as follows:

 $$\begin{array}{c} p_{2}^{\prime }=m^{\prime }u_{y}^{\prime }=\frac{m_{0}u_{y}^{\prime }}{\sqrt{1-\left({u^{\prime }}^{2}/c^{2}\right)}}. \end{array}$$

Having taken Equations (73) and (89) into account, we obtain

(95) $$\begin{array}{c} p_{2}^{\prime }=\frac{m_{0}\left(1-\left(uv/c^{2}\right)\right)u_{y}^{\prime }}{\sqrt{\left(1-\left(u^{2}/c^{2}\right)\right)\left(1-\left(v^{2}/c^{2}\right)\right)}},\\ p_{2}^{\prime }=\frac{m_{0}\left(1-\left(uv/c^{2}\right)\right)}{\sqrt{\left(1-\left(u^{2}/c^{2}\right)\right)\left(1-\left(v^{2}/c^{2}\right)\right)}}\times \frac{\left(u_{y}-v\sin\theta \right)}{\left(1-\left(uv/c^{2}\right)\right)},\\ p_{2}^{\prime }=\frac{m_{0}\left(u_{y}-v\sin\theta \right)}{\sqrt{\left(1-\left(u^{2}/c^{2}\right)\right)\left(1-\left(v^{2}/c^{2}\right)\right)}}=\frac{mu_{y}-mv\sin\theta }{\sqrt{1-\left(v^{2}/c^{2}\right)}},\\ p_{2}^{\prime }=\frac{p_{2}-\left(Ev\sin\theta /c^{2}\right)}{\sqrt{1-\left(v^{2}/c^{2}\right)}}. \end{array}$$

Equations (94) and (95) are the Lorentz transformation equations for the *X* and *Y*-components of linear momentum between inertial frames of reference moving in 2D *XY*-plane. After rearranging Equation (94) and having taken Equation (81) into account, we obtain

(96) $$\begin{array}{c} p_{1}^{\prime }=\frac{p_{1}-\left(Ev\cos\theta /c^{2}\right)}{\sqrt{1-\left(v^{2}/c^{2}\right)}}=\gamma \left(p_{1}+\frac{i^{2}Ev\cos\theta }{c^{2}}\right),\\ p_{1}^{\prime }=\gamma \left(p_{1}+\frac{i^{2}\beta E\cos\theta }{c}\right),\\ p_{1}^{\prime }=\gamma \left(p_{1}+i\beta p_{3}\right). \end{array}$$

Similarly, after rearranging Equation (95) and having taken Equation (81) into account, we obtain

(97) $$\begin{array}{c} p_{2}^{\prime }=\frac{p_{2}-\left(Ev\sin\theta /c^{2}\right)}{\sqrt{1-\left(v^{2}/c^{2}\right)}}=\gamma \left(p_{2}+\frac{i^{2}Ev\sin\theta }{c^{2}}\right),\\ p_{2}^{\prime }=\gamma \left(p_{2}+\frac{i^{2}\beta E\sin\theta }{c}\right),\\ p_{2}^{\prime }=\gamma \left(p_{2}+i\beta p_{4}\right). \end{array}$$

Also, after rearranging Equation (92), we obtain

$$\begin{array}{cc} \; & E^{\prime }=\frac{E-pv}{\sqrt{1-\left(v^{2}/c^{2}\right)}}=\gamma \left(E-pv\right),\\ \frac{\text{i}E^{\prime }\cos\theta }{c}=\gamma \left(\frac{iE\cos\theta }{c}-i\beta p\cos\theta \right). \end{array}$$

Having taken Equations (53), (81), and (82) into account, we obtain

(98) $$\begin{array}{c} p_{3}^{\prime }=\gamma \left(p_{3}-i\beta p_{1}\right). \end{array}$$

Also, after rearranging Equation (92), we obtain

$$\begin{array}{cc} \; & E^{\prime }=\frac{E-pv}{\sqrt{1-\left(v^{2}/c^{2}\right)}}=\gamma \left(E-pv\right),\\ \frac{iE^{\prime }\sin\theta }{c}=\gamma \left(\frac{iE\sin\theta }{c}-i\beta p\sin\theta \right). \end{array}$$

Having taken Equations (54), (81), and (82) into account, we obtain

(99) $$\begin{array}{c} p_{4}^{\prime }=\gamma \left(p_{4}-i\beta p_{2}\right). \end{array}$$

Formulas presented in Equations (96), (97), (98), and (99) are exactly the same as given in Equation (83). We had derived the transformation Equation (83) with the direct use of matrix equation of 2D Lorentz transformation equations, while in Equations (96), (97), (98), and (99) we have again exactly derived the same transformation equations with the use of the 2D Lorentz transformation equations.

## 6. Hyperbolic Form of Lorentz Transformations in 2 + 2 Dimensions

Now we seek to produce the hyperbolic form of 2D Lorentz transformation equations according to the invariance of spacetime interval Equation (13):

(100) $$\begin{array}{c} r^{2}-{\left(ct\right)}^{2}={r^{\prime }}^{2}-{\left(ct^{\prime }\right)}^{2}. \end{array}$$

Assume the transformation is linear and homogeneous, that is,

(101) $$\begin{array}{c} \left\{\begin{array}{c} r^{\prime }=mr+n\left(ct\right)\\ \left(ct^{\prime }\right)=fr+g\left(ct\right) \end{array}\right., \end{array}$$

where the coefficients *f*, *g*, *m*, and *n* are to be determined. Inserting Equation (101) into (100), we find

$$\begin{array}{cc} \; & r^{2}-{\left(ct\right)}^{2}={r^{\prime }}^{2}-{\left(ct^{\prime }\right)}^{2},\\ r^{2}-{\left(ct\right)}^{2}={\left[mr+n\left(ct\right)\right]}^{2}-{\left[fr+g\left(ct\right)\right]}^{2},\\ r^{2}-{\left(ct\right)}^{2}=\left(m^{2}-f^{2}\right)r^{2}-\left(g^{2}-n^{2}\right){\left(ct\right)}^{2}+2\left(mn-fg\right)r\left(ct\right). \end{array}$$

By equating the corresponding terms of *r*^2^, (*ct*)^2^, and *r*(*ct*), we obtain

(102) $$\begin{array}{c} \left\{\begin{array}{c} m^{2}-f^{2}=1\\ g^{2}-n^{2}=1\\ mn-fg=0 \end{array}\right.. \end{array}$$

The first equation in (102) suggests that *m* and *f* can be denoted by hyperbolic functions, without loss of generality, as

(103) $$\begin{array}{c} \left\{\begin{array}{c} m=\cosh\rho \\ f=\sinh\rho \end{array}\right., \end{array}$$

where *ρ* is an arbitrary parameter. Similarly, the second equation in (102) suggests that

(104) $$\begin{array}{c} \left\{\begin{array}{c} n=\sinh\psi \\ g=\cosh\psi \end{array}\right., \end{array}$$

where *ψ* is another arbitrary parameter. Inserting Equations (103) and (104) into the last equation of (102), one obtains

$$\begin{array}{cc} \; & \cosh\rho \sinh\psi -\sinh\rho \cosh\psi =0,\\ \sinh\left(\psi -\rho \right)=0,\\ \psi =\rho . \end{array}$$

Now the transformation (101) is simply denoted by

 $$\begin{array}{c} \left\{\begin{array}{c} ct^{\prime }=r\sinh\rho +ct\cosh\rho \end{array}\right., \end{array}$$

which is equivalent to

(105) $$\begin{array}{c} \left\{\begin{array}{c} r^{\prime }=r\cosh\rho -i^{2}ct\sinh\rho \\ ict^{\prime }=ri\sinh\rho +ict\cosh\rho \end{array}\right.. \end{array}$$

We can package this equation into a 2 × 2 matrix equation as follows:

(106) $$\begin{array}{c} \left[\begin{array}{c} r^{\prime }\\ {ict}^{\prime } \end{array}\right]=\left[\begin{array}{cc} \cosh\rho & -i\sinh\rho \\ i\sinh\rho & \cosh\rho \end{array}\right]\left[\begin{array}{c} r\\ ict \end{array}\right]. \end{array}$$

This equation represents the hyperbolic form of the Lorentz transformation equations of resultant spacetime coordinates. Now, from Equation (20), we have

 $$\begin{array}{c} r^{\prime }=\frac{r-vt}{\sqrt{1-\left(v^{2}/c^{2}\right)}}. \end{array}$$

Here when *r*′ = 0, then *r* = *vt*. Now, with these values, the first equation of (105) takes the form

$$\begin{array}{cc} \; & 0=vt\cosh\rho +ct\sinh\rho ,\\ \tanh\rho =-\frac{v}{c}=-\beta . \end{array}$$

Furthermore,

(107) $$\begin{array}{c} \left\{\begin{array}{c} \cosh\rho =\frac{1}{\sqrt{1-\tanh^{2}\rho }}=\frac{1}{\sqrt{1-\beta^{2}}}=\gamma \\ \sinh\rho =\cosh\rho \times \tanh\rho =-\beta \gamma \end{array}\right.. \end{array}$$

With the use of Equation (107) into (106), we obtain

 $$\begin{array}{c} \left[\begin{array}{c} {ict}^{\prime } \end{array}\right]=\left[\begin{array}{cc} i\beta \gamma \\ -i\beta \gamma & \gamma \end{array}\right]\left[\begin{array}{c} ict \end{array}\right]. \end{array}$$

This equation is exactly same as Equation (30). Now our main task is to obtain the hyperbolic form of Lorentz transformation for the components of spacetime coordinates. For this, multiplying the first equation in (105) by cos*θ*, we obtain

 $$\begin{array}{c} r^{\prime }\cos\theta =r\cos\theta \cosh\rho -i^{2}ct\cos\theta \sinh\rho . \end{array}$$

Having taken Equations (32), (34), and (38) into account, we obtain

(108) $$\begin{array}{c} z_{1}^{\prime }=z_{1}\cosh\rho -z_{3}i\sinh\rho . \end{array}$$

In the same way, multiplying the first equation in (105) by sin*θ* and having taken Equations (33), (35), and (39) into account, we obtain

(109) $$\begin{array}{c} r^{\prime }\sin\theta =r\sin\theta \cosh\rho -i^{2}ct\sin\theta \sinh\rho ,\\ z_{2}^{\prime }=z_{2}\cosh\rho -z_{4}i\sinh\rho . \end{array}$$

Similarly, multiplying the second equation in (105) by cos*θ*, we obtain

 $$\begin{array}{c} ict^{\prime }\cos\theta =r\cos\theta i\sinh\rho +ict\cos\theta \cosh\rho . \end{array}$$

Having taken Equations (32), (38), and (40) into account, we obtain

(110) $$\begin{array}{c} z_{3}^{\prime }=z_{1}i\sinh\rho +z_{3}\cosh\rho . \end{array}$$

In the same way, multiplying the second equation in (105) by sin*θ* and having taken Equations (33), (39), and (41) into account, we obtain

(111) $$\begin{array}{c} ict^{\prime }\sin\theta =r\sin\theta i\sinh\rho +ict\sin\theta \cosh\rho ,\\ z_{4}^{\prime }=z_{2}i\sinh\rho +z_{4}\cosh\rho . \end{array}$$

Finally, we can package the Lorentz transformation Equations (108), (109), (110), and (111) into a 4 × 4  matrix form as follows:

(112) $$\begin{array}{c} \left[\begin{array}{c} z_{1}^{\prime }\\ z_{2}^{\prime }\\ \begin{array}{c} z_{3}^{\prime }\\ z_{4}^{\prime } \end{array} \end{array}\right]=\left[\begin{array}{cccc} \cosh\rho & 0 & -i\sinh\rho & 0\\ 0 & \cosh\rho & 0 & -i\sinh\rho \\ i\sinh\rho & 0 & \cosh\rho & 0\\ 0 & i\sinh\rho & 0 & \cosh\rho \end{array}\right]\left[\begin{array}{c} z_{1}\\ z_{2}\\ z_{3}\\ z_{4} \end{array}\right]. \end{array}$$

This expression represents the matrix equation of 2D Lorentz transformation equations in hyperbolic form. Also, with the use of Equation (107) into (112), we obtain the following matrix equation, which is exactly the same as Equation (47):

 $$\begin{array}{c} \left[\begin{array}{c} z_{2}^{\prime }\\ \begin{array}{c} z_{4}^{\prime } \end{array} \end{array}\right]=\left[\begin{array}{cccc} 0 & i\beta \gamma & 0\\ 0 & \gamma & 0 & i\beta \gamma \\ -i\beta \gamma & 0 & \gamma & 0\\ 0 & -i\beta \gamma & 0 & \gamma \end{array}\right]\left[\begin{array}{c} z_{2}\\ z_{3}\\ z_{4} \end{array}\right]. \end{array}$$

## 7. Invariance of the D'Alembert Operator

The D'Alembert operator (denoted by a box: □) in terms of resultant radius vector *r* can be defined as follows:

 $$\begin{array}{c} □=\frac{\partial^{2}}{\partial r^{2}}-\frac{\partial^{2}}{\partial {\left(ct\right)}^{2}}=\frac{\partial^{2}}{\partial r^{2}}+\frac{\partial^{2}}{\partial {\left(ict\right)}^{2}}. \end{array}$$

In 2D *XY*-plane, radius vector *r* has two components *z*_1_ = *x* and *z*_2_ = *y*. Hence, the foregoing equation in terms of the components of *r* can be expanded as follows:

 $$\begin{array}{c} □=\frac{\partial^{2}}{\partial x^{2}}+\frac{\partial^{2}}{\partial y^{2}}+\frac{\partial^{2}}{\partial {\left(ict\right)}^{2}}=\frac{\partial^{2}}{\partial {\left(\;z_{1}\right)}^{2}}+\frac{\partial^{2}}{\partial {\left(\;z_{2}\right)}^{2}}+\frac{\partial^{2}}{\partial {\left(ict\right)}^{2}}. \end{array}$$

In 2 + 2-dimensional spacetime continuum, *ict* has two components, namely, (*z*_3_, *z*_4_ ). Hence, the foregoing equation in terms of the components of *ict* can be expanded as follows:

(113) $$\begin{array}{c} □=\frac{\partial^{2}}{\partial {\left(\;z_{1}\right)}^{2}}+\frac{\partial^{2}}{\partial {\left(\;z_{2}\right)}^{2}}+\frac{\partial^{2}}{\partial {\left(\;z_{3}\right)}^{2}}+\frac{\partial^{2}}{\partial {\left(\;z_{4}\right)}^{2}}. \end{array}$$

Equation (113) represents the expression of the D'Alembert operator in 2 + 2 dimensions of spacetime continuum. Similarly, the D'Alembert operator in frame *S*′ would be in the following form:

(114) $$\begin{array}{c} {□}^{\prime }=\frac{\partial^{2}}{\partial {\left(z_{1}^{\prime }\right)}^{2}}+\frac{\partial^{2}}{\partial {\left(z_{2}^{\prime }\right)}^{2}}+\frac{\partial^{2}}{\partial {\left(z_{3}^{\prime }\right)}^{2}}+\frac{\partial^{2}}{\partial {\left(z_{4}^{\prime }\right)}^{2}}. \end{array}$$

In order to prove the invariance of the D'Alembert operator, let us consider an electromagnetic wave is travelling in system *S*, and then, the propagation equation for such a wave using Equation (113) is of the form

 $$\begin{array}{c} \left(\frac{\partial^{2}}{\partial {\left(\;z_{1}\right)}^{2}}+\frac{\partial^{2}}{\partial {\left(\;z_{2}\right)}^{2}}+\frac{\partial^{2}}{\partial {\left(\;z_{3}\right)}^{2}}+\frac{\partial^{2}}{\partial {\left(\;z_{4}\right)}^{2}}\right)\psi =0. \end{array}$$

Here *ψ* is the wavefunction and it clearly depends on the parameters *z*_1_, *z*_2_, *z*_3_, and *z*_4_, and thus, it can be written as *ψ*(*z*_1_, *z*_2_, *z*_3_, *z*_4_ ). Now in frame *S*′, which is moving relative to frame *S*, the propagation equation of the same wave using Equation (114) is given by

 $$\begin{array}{c} \left(\frac{\partial^{2}}{\partial {\left(z_{1}^{\prime }\right)}^{2}}+\frac{\partial^{2}}{\partial {\left(z_{2}^{\prime }\right)}^{2}}+\frac{\partial^{2}}{\partial {\left(z_{3}^{\prime }\right)}^{2}}+\frac{\partial^{2}}{\partial {\left(z_{4}^{\prime }\right)}^{2}}\right)\psi^{\prime }=0. \end{array}$$

Thus, *ψ*′ may be written as

 $$\begin{array}{c} \psi^{\prime }\left(z_{1}^{\prime },z_{2}^{\prime },z_{3}^{\prime },z_{4}^{\prime }\right). \end{array}$$

We thus have

$$\begin{array}{cc} \; & \frac{\partial \psi^{\prime }}{\partial z_{1}}=\frac{\partial \psi^{\prime }}{\partial z_{1}^{\prime }}\frac{\partial z_{1}^{\prime }}{\partial z_{1}}+\frac{\partial \psi^{\prime }}{\partial z_{2}^{\prime }}\frac{\partial z_{2}^{\prime }}{\partial z_{1}}+\frac{\partial \psi^{\prime }}{\partial z_{3}^{\prime }}\frac{\partial z_{3}^{\prime }}{\partial z_{1}}+\frac{\partial \psi^{\prime }}{\partial z_{4}^{\prime }}\frac{\partial z_{4}^{\prime }}{\partial z_{1}},\\ \frac{\partial }{\partial z_{1}}=\frac{\partial }{\partial z_{1}^{\prime }}\frac{\partial z_{1}^{\prime }}{\partial z_{1}}+\frac{\partial }{\partial z_{2}^{\prime }}\frac{\partial z_{2}^{\prime }}{\partial z_{1}}+\frac{\partial }{\partial z_{3}^{\prime }}\frac{\partial z_{3}^{\prime }}{\partial z_{1}}+\frac{\partial }{\partial z_{4}^{\prime }}\frac{\partial z_{4}^{\prime }}{\partial z_{1}}. \end{array}$$

Next, making use of Equations (108), (109), (110), and (111), the foregoing equation can be written as

$$\begin{array}{cc} \; & \frac{\partial }{\partial z_{1}}=\frac{\partial }{\partial z_{1}^{\prime }}\frac{\partial \left(z_{1}\cosh\rho -z_{3}i\sinh\rho \right)}{\partial z_{1}}+\frac{\partial }{\partial z_{2}^{\prime }}\frac{\partial \left(z_{2}\cosh\rho -z_{4}i\sinh\rho \right)}{\partial z_{1}}+\frac{\partial }{\partial z_{3}^{\prime }}\frac{\partial \left(z_{1}i\sinh\rho +z_{3}\cosh\rho \right)}{\partial z_{1}}+\frac{\partial }{\partial z_{4}^{\prime }}\frac{\partial \left(z_{2}i\sinh\rho +z_{4}\cosh\rho \right)}{\partial z_{1}},\\ \frac{\partial }{\partial z_{1}}=\frac{\partial }{\partial z_{1}^{\prime }}\cosh\rho +\frac{\partial }{\partial z_{2}^{\prime }}0+\frac{\partial }{\partial z_{3}^{\prime }}i\sinh\rho +\frac{\partial }{\partial z_{4}^{\prime }}0,\\ \frac{\partial }{\partial z_{1}}=\cosh\rho \frac{\partial }{\partial z_{1}^{\prime }}+i\sinh\rho \frac{\partial }{\partial z_{3}^{\prime }}. \end{array}$$

Multiplying the above equation by itself, we get

(115) $$\begin{array}{c} \frac{\partial^{2}}{\partial {\left(\;z_{1}\right)}^{2}}=\left(\cosh\rho \frac{\partial }{\partial z_{1}^{\prime }}+i\sinh\rho \frac{\partial }{\partial z_{3}^{\prime }}\right)\left(\cosh\rho \frac{\partial }{\partial z_{1}^{\prime }}+i\sinh\rho \frac{\partial }{\partial z_{3}^{\prime }}\right),\\ \frac{\partial^{2}}{\partial {\left(\;z_{1}\right)}^{2}}=\cosh^{2}\rho \frac{\partial^{2}}{\partial {\left(z_{1}^{\prime }\right)}^{2}}+2i\cosh\rho \sinh\rho \frac{\partial }{\partial z_{1}^{\prime }}\frac{\partial }{\partial z_{3}^{\prime }}-\sinh^{2}\rho \frac{\partial^{2}}{\partial {\left(z_{3}^{\prime }\right)}^{2}}. \end{array}$$

In the same way, we can write the following differential operator:

 $$\begin{array}{c} \frac{\partial }{\partial z_{3}}=\frac{\partial }{\partial z_{1}^{\prime }}\frac{\partial z_{1}^{\prime }}{\partial z_{3}}+\frac{\partial }{\partial z_{2}^{\prime }}\frac{\partial z_{2}^{\prime }}{\partial z_{3}}+\frac{\partial }{\partial z_{3}^{\prime }}\frac{\partial z_{3}^{\prime }}{\partial z_{3}}+\frac{\partial }{\partial z_{4}^{\prime }}\frac{\partial z_{4}^{\prime }}{\partial z_{3}}. \end{array}$$

Next, making use of Equations (108), (109), (110), and (111), the foregoing equation can be written as

$$\begin{array}{cc} \; & \frac{\partial }{\partial z_{3}}=\frac{\partial }{\partial z_{1}^{\prime }}\frac{\partial \left(z_{1}\cosh\rho -z_{3}i\sinh\rho \right)}{\partial z_{3}}+\frac{\partial }{\partial z_{2}^{\prime }}\frac{\partial \left(z_{2}\cosh\rho -z_{4}i\sinh\rho \right)}{\partial z_{3}}+\frac{\partial }{\partial z_{3}^{\prime }}\frac{\partial \left(z_{1}i\sinh\rho +z_{3}\cosh\rho \right)}{\partial z_{3}}+\frac{\partial }{\partial z_{4}^{\prime }}\frac{\partial \left(z_{2}i\sinh\rho +z_{4}\cosh\rho \right)}{\partial z_{3}},\\ \frac{\partial }{\partial z_{3}}=-i\sinh\rho \frac{\partial }{\partial z_{1}^{\prime }}+\frac{\partial }{\partial z_{2}^{\prime }}0+\cosh\rho \frac{\partial }{\partial z_{3}^{\prime }}+\frac{\partial }{\partial z_{4}^{\prime }}0,\\ \frac{\partial }{\partial z_{3}}=-i\sinh\rho \frac{\partial }{\partial z_{1}^{\prime }}+\cosh\rho \frac{\partial }{\partial z_{3}^{\prime }}. \end{array}$$

Multiplying the above equation by itself, we get

(116) $$\begin{array}{c} \frac{\partial^{2}}{\partial {\left(\;z_{3}\right)}^{2}}=\left(-i\sinh\rho \frac{\partial }{\partial z_{1}^{\prime }}+\cosh\rho \frac{\partial }{\partial z_{3}^{\prime }}\right)\left(-i\sinh\rho \frac{\partial }{\partial z_{1}^{\prime }}+\cosh\rho \frac{\partial }{\partial z_{3}^{\prime }}\right),\\ \frac{\partial^{2}}{\partial {\left(\;z_{3}\right)}^{2}}=-\sinh^{2}\rho \frac{\partial^{2}}{\partial {\left(z_{1}^{\prime }\right)}^{2}}-2i\cosh\rho \sinh\rho \frac{\partial }{\partial z_{1}^{\prime }}\frac{\partial }{\partial z_{3}^{\prime }}+\cosh^{2}\rho \frac{\partial^{2}}{\partial {\left(z_{3}^{\prime }\right)}^{2}}. \end{array}$$

Adding Equations (115) and (116) results in

(117) $$\begin{array}{c} \frac{\partial^{2}}{\partial {\left(\;z_{1}\right)}^{2}}+\frac{\partial^{2}}{\partial {\left(\;z_{3}\right)}^{2}}=\cosh^{2}\rho \frac{\partial^{2}}{\partial {\left(z_{1}^{\prime }\right)}^{2}}-\sinh^{2}\rho \frac{\partial^{2}}{\partial {\left(z_{3}^{\prime }\right)}^{2}}-\sinh^{2}\rho \frac{\partial^{2}}{\partial {\left(z_{1}^{\prime }\right)}^{2}}+\cosh^{2}\rho \frac{\partial^{2}}{\partial {\left(z_{3}^{\prime }\right)}^{2}},\\ \frac{\partial^{2}}{\partial {\left(\;z_{1}\right)}^{2}}+\frac{\partial^{2}}{\partial {\left(\;z_{3}\right)}^{2}}=\left(\cosh^{2}\rho -\sinh^{2}\rho \right)\frac{\partial^{2}}{\partial {\left(z_{1}^{\prime }\right)}^{2}}+\left(\cosh^{2}\rho -\sinh^{2}\rho \right)\frac{\partial^{2}}{\partial {\left(z_{3}^{\prime }\right)}^{2}},\\ \frac{\partial^{2}}{\partial {\left(\;z_{1}\right)}^{2}}+\frac{\partial^{2}}{\partial {\left(\;z_{3}\right)}^{2}}=\frac{\partial^{2}}{\partial {\left(z_{1}^{\prime }\right)}^{2}}+\frac{\partial^{2}}{\partial {\left(z_{3}^{\prime }\right)}^{2}}. \end{array}$$

Similarly, we can write the following differential operator:

 $$\begin{array}{c} \frac{\partial }{\partial z_{2}}=\frac{\partial }{\partial z_{1}^{\prime }}\frac{\partial z_{1}^{\prime }}{\partial z_{2}}+\frac{\partial }{\partial z_{2}^{\prime }}\frac{\partial z_{2}^{\prime }}{\partial z_{2}}+\frac{\partial }{\partial z_{3}^{\prime }}\frac{\partial z_{3}^{\prime }}{\partial z_{2}}+\frac{\partial }{\partial z_{4}^{\prime }}\frac{\partial z_{4}^{\prime }}{\partial z_{2}}. \end{array}$$

Next, making use of Equations (108), (109), (110), and (111), the foregoing equation can be written as

$$\begin{array}{cc} \; & \frac{\partial }{\partial z_{2}}=\frac{\partial }{\partial z_{1}^{\prime }}\frac{\partial \left(z_{1}\cosh\rho -z_{3}i\sinh\rho \right)}{\partial z_{2}}+\frac{\partial }{\partial z_{2}^{\prime }}\frac{\partial \left(z_{2}\cosh\rho -z_{4}i\sinh\rho \right)}{\partial z_{2}}+\frac{\partial }{\partial z_{3}^{\prime }}\frac{\partial \left(z_{1}i\sinh\rho +z_{3}\cosh\rho \right)}{\partial z_{2}}+\frac{\partial }{\partial z_{4}^{\prime }}\frac{\partial \left(z_{2}i\sinh\rho +z_{4}\cosh\rho \right)}{\partial z_{2}},\\ \frac{\partial }{\partial z_{2}}=\frac{\partial }{\partial z_{1}^{\prime }}0+\frac{\partial }{\partial z_{2}^{\prime }}\cosh\rho +\frac{\partial }{\partial z_{3}^{\prime }}0+\frac{\partial }{\partial z_{4}^{\prime }}i\sinh\rho ,\\ \frac{\partial }{\partial z_{2}}=\cosh\rho \frac{\partial }{\partial z_{2}^{\prime }}+i\sinh\rho \frac{\partial }{\partial z_{4}^{\prime }}. \end{array}$$

Multiplying the above equation by itself, we get

(118) $$\begin{array}{c} \frac{\partial^{2}}{\partial {\left(\;z_{2}\right)}^{2}}=\left(\cosh\rho \frac{\partial }{\partial z_{2}^{\prime }}+i\sinh\rho \frac{\partial }{\partial z_{4}^{\prime }}\right)\left(\cosh\rho \frac{\partial }{\partial z_{2}^{\prime }}+i\sinh\rho \frac{\partial }{\partial z_{4}^{\prime }}\right),\\ \frac{\partial^{2}}{\partial {\left(\;z_{2}\right)}^{2}}=\cosh^{2}\rho \frac{\partial^{2}}{\partial {\left(z_{2}^{\prime }\right)}^{2}}+2i\cosh\rho \sinh\rho \frac{\partial }{\partial z_{2}^{\prime }}\frac{\partial }{\partial z_{4}^{\prime }}-\sinh^{2}\rho \frac{\partial^{2}}{\partial {\left(z_{4}^{\prime }\right)}^{2}}. \end{array}$$

In the same way, we can write the following differential operator:

 $$\begin{array}{c} \frac{\partial }{\partial z_{4}}=\frac{\partial }{\partial z_{1}^{\prime }}\frac{\partial z_{1}^{\prime }}{\partial z_{4}}+\frac{\partial }{\partial z_{2}^{\prime }}\frac{\partial z_{2}^{\prime }}{\partial z_{4}}+\frac{\partial }{\partial z_{3}^{\prime }}\frac{\partial z_{3}^{\prime }}{\partial z_{4}}+\frac{\partial }{\partial z_{4}^{\prime }}\frac{\partial z_{4}^{\prime }}{\partial z_{4}}. \end{array}$$

Next, making use of Equations (108), (109), (110), and (111), the foregoing equation can be written as

$$\begin{array}{cc} \; & \frac{\partial }{\partial z_{4}}=\frac{\partial }{\partial z_{1}^{\prime }}\frac{\partial \left(z_{1}\cosh\rho -z_{3}i\sinh\rho \right)}{\partial z_{4}}+\frac{\partial }{\partial z_{2}^{\prime }}\frac{\partial \left(z_{2}\cosh\rho -z_{4}i\sinh\rho \right)}{\partial z_{4}}+\frac{\partial }{\partial z_{3}^{\prime }}\frac{\partial \left(z_{1}i\sinh\rho +z_{3}\cosh\rho \right)}{\partial z_{4}}+\frac{\partial }{\partial z_{4}^{\prime }}\frac{\partial \left(z_{2}i\sinh\rho +z_{4}\cosh\rho \right)}{\partial z_{4}},\\ \frac{\partial }{\partial z_{4}}=0\frac{\partial }{\partial z_{1}^{\prime }}-i\sinh\rho \frac{\partial }{\partial z_{2}^{\prime }}+0\frac{\partial }{\partial z_{3}^{\prime }}+\cosh\rho \frac{\partial }{\partial z_{4}^{\prime }},\\ \frac{\partial }{\partial z_{4}}=-i\sinh\rho \frac{\partial }{\partial z_{2}^{\prime }}+\cosh\rho \frac{\partial }{\partial z_{4}^{\prime }}. \end{array}$$

Multiplying the above equation by itself, we get

(119) $$\begin{array}{c} \frac{\partial^{2}}{\partial {\left(\;z_{4}\right)}^{2}}=\left(-i\sinh\rho \frac{\partial }{\partial z_{2}^{\prime }}+\cosh\rho \frac{\partial }{\partial z_{4}^{\prime }}\right)\left(-i\sinh\rho \frac{\partial }{\partial z_{2}^{\prime }}+\cosh\rho \frac{\partial }{\partial z_{4}^{\prime }}\right),\\ \frac{\partial^{2}}{\partial {\left(\;z_{4}\right)}^{2}}=-\sinh^{2}\rho \frac{\partial^{2}}{\partial {\left(z_{2}^{\prime }\right)}^{2}}-2i\cosh\rho \sinh\rho \frac{\partial }{\partial z_{2}^{\prime }}\frac{\partial }{\partial z_{4}^{\prime }}+\cosh^{2}\rho \frac{\partial^{2}}{\partial {\left(z_{4}^{\prime }\right)}^{2}}. \end{array}$$

Adding Equations (118) and (119) results in

(120) $$\begin{array}{c} \frac{\partial^{2}}{\partial {\left(\;z_{2}\right)}^{2}}+\frac{\partial^{2}}{\partial {\left(\;z_{4}\right)}^{2}}=\cosh^{2}\rho \frac{\partial^{2}}{\partial {\left(z_{2}^{\prime }\right)}^{2}}-\sinh^{2}\rho \frac{\partial^{2}}{\partial {\left(z_{4}^{\prime }\right)}^{2}}-\sinh^{2}\rho \frac{\partial^{2}}{\partial {\left(z_{2}^{\prime }\right)}^{2}}+\cosh^{2}\rho \frac{\partial^{2}}{\partial {\left(z_{4}^{\prime }\right)}^{2}},\\ \frac{\partial^{2}}{\partial {\left(\;z_{2}\right)}^{2}}+\frac{\partial^{2}}{\partial {\left(\;z_{4}\right)}^{2}}=\left(\cosh^{2}\rho -\sinh^{2}\rho \right)\frac{\partial^{2}}{\partial {\left(z_{2}^{\prime }\right)}^{2}}+\left(\cosh^{2}\rho -\sinh^{2}\rho \right)\frac{\partial^{2}}{\partial {\left(z_{4}^{\prime }\right)}^{2}},\\ \frac{\partial^{2}}{\partial {\left(\;z_{2}\right)}^{2}}+\frac{\partial^{2}}{\partial {\left(\;z_{4}\right)}^{2}}=\frac{\partial^{2}}{\partial {\left(z_{2}^{\prime }\right)}^{2}}+\frac{\partial^{2}}{\partial {\left(z_{4}^{\prime }\right)}^{2}}. \end{array}$$

Adding Equations (117) and (120) results in

(121) $$\begin{array}{c} \frac{\partial^{2}}{\partial {\left(\;z_{1}\right)}^{2}}+\frac{\partial^{2}}{\partial {\left(\;z_{2}\right)}^{2}}+\frac{\partial^{2}}{\partial {\left(\;z_{3}\right)}^{2}}+\frac{\partial^{2}}{\partial {\left(\;z_{4}\right)}^{2}}=\frac{\partial^{2}}{\partial {\left(z_{1}^{\prime }\right)}^{2}}+\frac{\partial^{2}}{\partial {\left(z_{2}^{\prime }\right)}^{2}}+\frac{\partial^{2}}{\partial {\left(z_{3}^{\prime }\right)}^{2}}+\frac{\partial^{2}}{\partial {\left(z_{4}^{\prime }\right)}^{2}}. \end{array}$$

Based on Equations (113), (114), and (121), we may say that the D'Alembert operator is invariant under the 2 + 2-dimensional Lorentz transformation equations.

## 8. Conclusions

We introduced two space and two time coordinates (2 + 2) to extend the classical one-dimensional Lorentz transformation equations to two dimensions to derive the relativistic transformation equations of physical quantities between inertial frames of references moving in 2D *XY*-plane. The concept of 2 + 2-dimensional spacetime continuum allows us to deduce the matrix form of these transformation equations. Based on the notion of 2 + 2-dimensional spacetime continuum, we have expressed the 2D Lorentz transformation equations in terms of four-vector, which have also been presented in hyperbolic forms. Additionally, we have achieved the Lorentz velocity addition, transformation of energy and momentum with the use of the derived 2D transformations which fully agree with conventional Lorentz invariant energy–momentum relations. We hope that the proposed study based on the concept of two space and two time coordinates will be useful in understanding mathematical features of the spacetime continuum between inertial frames of reference moving in a 2D *XY*-plane.

## Figures and Tables

**Table 1:** Lorentz transformation equations along *X* and *Y*-directions.

**Motion between frames**	**Transformation of ** **X** ** -coordinate**	**Transformation of ** **Y** ** -coordinate**	**Transformation of time**
Along *X* and *Y*-directions	From Equation (23), $x^{\prime }=x\left(1-\left(vt/\sqrt{x^{2}+y^{2}}\right)\right)/\sqrt{1-\left(v^{2}/c^{2}\right)}$	From Equation (25), $y^{\prime }=y\left(1-\left(vt/\sqrt{x^{2}+y^{2}}\right)\right)/\sqrt{1-\left(v^{2}/c^{2}\right)}$	From Equation (26), $t^{\prime }=t-\left(v\sqrt{x^{2}+y^{2}}/c^{2}\right)/\sqrt{1-\left(v^{2}/c^{2}\right)}$
Along *X*-direction *y* = *y*′ = 0	$x^{\prime }=\frac{x\left(1-\left(vt/\sqrt{x^{2}+0^{2}}\right)\right)}{\sqrt{1-\left(v^{2}/c^{2}\right)}}$ $x^{\prime }=\frac{x-vt}{\sqrt{1-\left(v^{2}/c^{2}\right)}}$	$y^{\prime }=\frac{0\left(1-\left(vt/\sqrt{x^{2}+y^{2}}\right)\right)}{\sqrt{1-\left(v^{2}/c^{2}\right)}}$ *y*′ = 0	$t^{\prime }=\frac{t-\left(v\sqrt{x^{2}+0^{2}}/c^{2}\right)}{\sqrt{1-\left(v^{2}/c^{2}\right)}}$ $t^{\prime }=\frac{t-\left(vx/c^{2}\right)}{\sqrt{1-\left(v^{2}/c^{2}\right)}}$
Along *Y*-direction *x* = *x*′ = 0	$x^{\prime }=\frac{0\left(1-\left(vt/\sqrt{x^{2}+y^{2}}\right)\right)}{\sqrt{1-\left(v^{2}/c^{2}\right)}}$ *x*′ = 0	$y^{\prime }=\frac{y\left(1-\left(vt/\sqrt{0^{2}+y^{2}}\right)\right)}{\sqrt{1-\left(v^{2}/c^{2}\right)}}$ $y^{\prime }=\frac{y-vt}{\sqrt{1-\left(v^{2}/c^{2}\right)}}$	$t^{\prime }=\frac{t-\left(v\sqrt{0^{2}+y^{2}}/c^{2}\right)}{\sqrt{1-\left(v^{2}/c^{2}\right)}}$ $t^{\prime }=\frac{t-\left(vy/c^{2}\right)}{\sqrt{1-\left(v^{2}/c^{2}\right)}}$

## Data Availability

Data sharing is not applicable—no new data are generated, or the article describes entirely theoretical research.
